# Antibacterial activity of structurally diverse natural prenylated isobavachalcone derivatives†

**DOI:** 10.1039/d4ra05370b

**Published:** 2024-10-17

**Authors:** Puneet Kumar, Sapna Saini, Anjali Gangwar, Rashmi Sharma, Jasha Momo H. Anal

**Affiliations:** a Natural Products and Medicinal Chemistry Division, CSIR– Indian Institute of Integrative Medicine Jammu 180001 India hmunshel.jasha@iiim.res.in; b Infectious Division, CSIR–Indian Institute of Integrative Medicine Jammu 180001 India rashmi.sharma.09@iiim.res.in; c Academy of Scientific and Innovative Research (AcSIR) Ghaziabad 201002 India

## Abstract

Isobavachalcone (IBC) is a natural prenylated flavonoid containing chalcone and prenyl chain moieties with a wide range of biological and pharmacological properties. In this work, we synthesized structurally diversified derivatives (IBC-2 to IBC-10) from the natural prenylated chalcone IBC isolated from *Psoralea corylifolia* and assessed their antibacterial potency against the Gram-positive and Gram-negative bacterial strains *S. aureus* ATCC 29213, *MRSA* ATCC 15187, *E. coli* ATCC25922 and *P. aeruginosa* ATCC 27853. IBC and IBC-2 exhibited a minimum inhibition concentration (MIC) of 5.0 μM against *S. aureus* ATCC 29213, whereas IBC-3 exhibited a broad-spectrum activity against Gram-positive and Gram-negative pathogens. Cytotoxicity assessments on the murine RAW 264.7 macrophage cell line revealed minimal to moderate cytotoxicity for IBC-2 and IBC-3 with a favorable selectivity index (>10). Time- and concentration-dependent studies further supported the bactericidal nature of the compounds, as IBC, IBC-2, and IBC-3 exhibited concentration-dependent killing of *S. aureus* in a time-dependent manner. Furthermore, combination studies, SEM analysis, and PI staining suggest that IBC-3's mechanism of action targets the bacteria's cytoplasmic membrane or cell wall. The bioactive compounds displayed promising drug-like characteristics and a favorable pharmacokinetic profile (ADME-Tox), indicating a projected high oral bioavailability. Structure–activity relationships (SARs) drawn from this study reveal that a prenyl chain at the A-ring and hydroxy functional groups attached to the aromatic rings of chalcone scaffolds are responsible for this antibacterial potential, which will be helpful in the future discovery and development of antibiotics from natural products to overcome the antibiotic resistance crisis.

## Abstract 2

Naturally occurring prenylated Isobavachalcone and derivatives show antibacterial properties against clinically significant pathogens and demonstrated robust antimicrobial activity.

## Graphical abstract

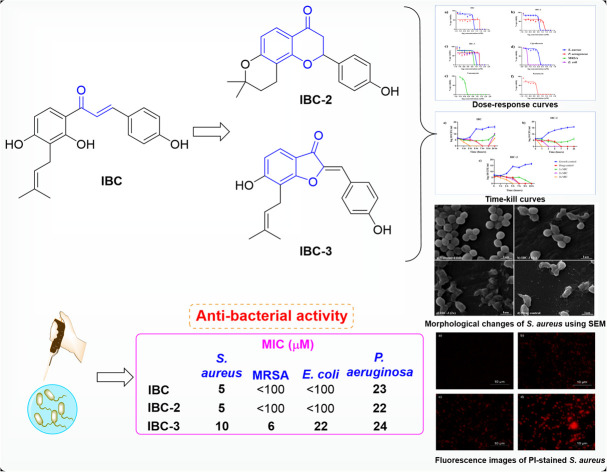

## 1. Introduction

Antimicrobial resistance (AMR) is a global health threat and concern. The causes and contributing factors vary owing to the overuse of existing antibiotics, genetic elements, control practices, environments, and unknown infection routes.^1^ The reason for concern about AMR is that resistant infections can be difficult and sometimes impossible to treat. In 2019, reports documented that at least 1.27 million people worldwide died because of AMR, and nearly 5 million deaths were associated with this resistant infection.^2,3^ Antimicrobial resistance threatens various sectors, impacting individuals at different life stages and affecting healthcare, veterinary, and agriculture industries. The resistance to a single antibiotic can lead to significant complications, such as severe side effects in infections requiring advanced treatments, jeopardizing medical procedures such as organ transplants and cancer therapies and the management of chronic conditions such as diabetes and rheumatoid arthritis. Infections may become untreatable in certain instances, emphasizing the critical need for addressing antimicrobial resistance.^3–5^ Natural products and their diverse scaffolds played a pivotal role in antibiotic drug discovery, with most antibacterial drugs derived from natural or natural product leads.^6^ Prenylated flavonoids are a unique class of naturally occurring phenolics characterized by a prenyl side chain in the flavonoid skeleton and exhibit diverse biological and pharmacological activities.^7^ In nature, flavonoids are abundant; however, prenylated flavonoids are much less common but have received attention considering their beneficial and massive scope of applications in the food industry and pharmaceuticals,^8^ and they are mainly isolated from plants belonging to the *Fabaceae*, *Leguminosae*, *Moraceae*, and *Canabaceae* families.^9^ Additionally, chalcones are simple chemical scaffolds found in many natural compounds. They are considered a privileged structure in medicinal chemistry not only from the synthetic and biosynthetic perspective but also due to their broad, interesting biological activities. They are characterized by a specific chemical structure consisting of two aromatic rings and a three-carbon bridge containing an α, β-unsaturated carbonyl group.^10^ Aromatic metabolites containing prenyl side chains represent a rare class of natural products with interesting and valuable bioactive constituents for decades.^11^ Prenylation of flavonoids is reported to increase their lipophilicity, affinity to biological membranes, and interactions with the target proteins, which results in enhanced biological activities such as antimicrobial and estrogenic activities.^7^ Today, the antibiotic crisis necessitates creative and innovative approaches, and natural products represent a promising source of antibacterial lead compounds for discovering novel classes.^12^

**Fig. 1:**
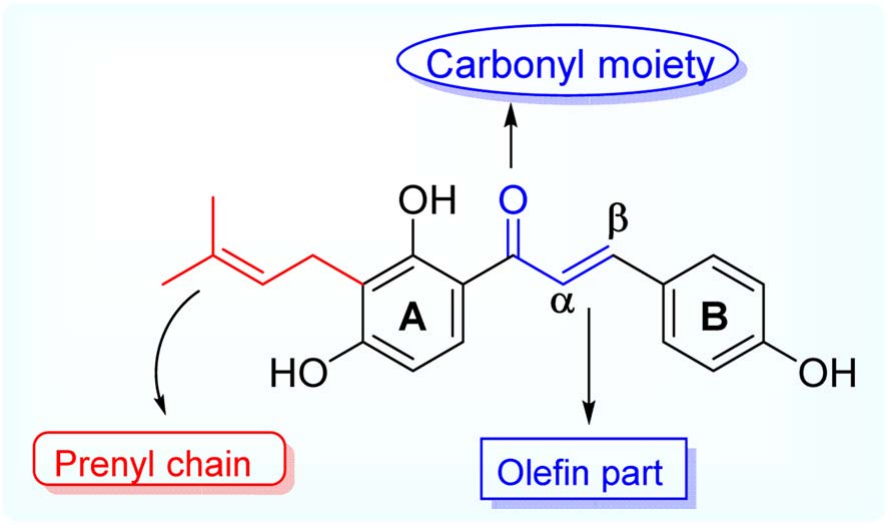
Representation of the prenylated natural product isobavachalcone (IBC).

Isobavachalcone (IBC), (*E*)-1-(2,4-dihydroxy-3-(3-methylbut-2-en-1-yl)phenyl)-3-(4-hydroxyphenyl) prop-2-en-1-one is one of the naturally occurring prenylated chalcone, first reported being isolated from *Psoralea corylifolia*, a plant belonging to the legume family (*Fabaceae*) whereby the dry fruits are used for its traditional medicinal purposes for treating various ailments like bone fractures, enuresis pollakiuria, osteomalacia and osteoporosis, waist and knee psychroalgia, and gynecological bleeding in India and China.^13^ Modern pharmacological studies have reported its biological activities, including anti-cancer, antibacterial, anti-inflammatory, *etc.*^14^ Researchers have been studying the biological impacts of flavonoids for decades, with an emphasis on significant subclasses such as chalcones, flavones, flavanones, and aurones.^15,16^ Natural prenylated chalcones have been reported to exhibit antibacterial properties.^17–19^ Various natural plant compounds, such as flavonoids, have also been noted for their potent antimicrobial activity. Aurones occur naturally in only a few advanced plant species, where they play significant roles. Compounds such as cephalocerone and hispidol have demonstrated antimicrobial properties.^20^ This study aims to isolate naturally occurring prenylated chalcone (IBC) and attempt cost-effective semi-synthetic modifications to obtain structurally diverse derivatives and access their antimicrobial potential (Fig. 1).

## 2. Results and discussions

### 2.1 Chemistry section

**Scheme 1:**
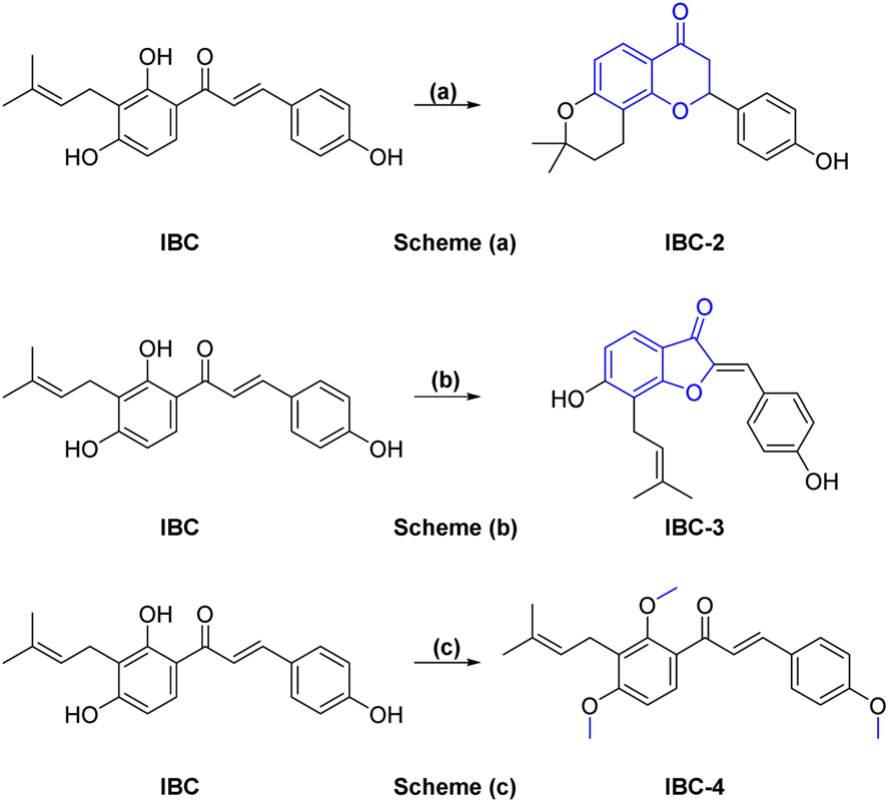
*Reagents and conditions*: (a) 10% aq. NaOH, MeOH : H_2_O (1 : 1), 4 h, rt, then 50% H_2_SO_4_ dropwise, yield 60%; (b) Hg(OAc)_2_, pyridine, reflux at 110 °C, 5 h, yield 70%; (c) CH_3_I, NaH, DMF, 0 °C–rt, 3 h, yield 75%.

**Scheme 2:**
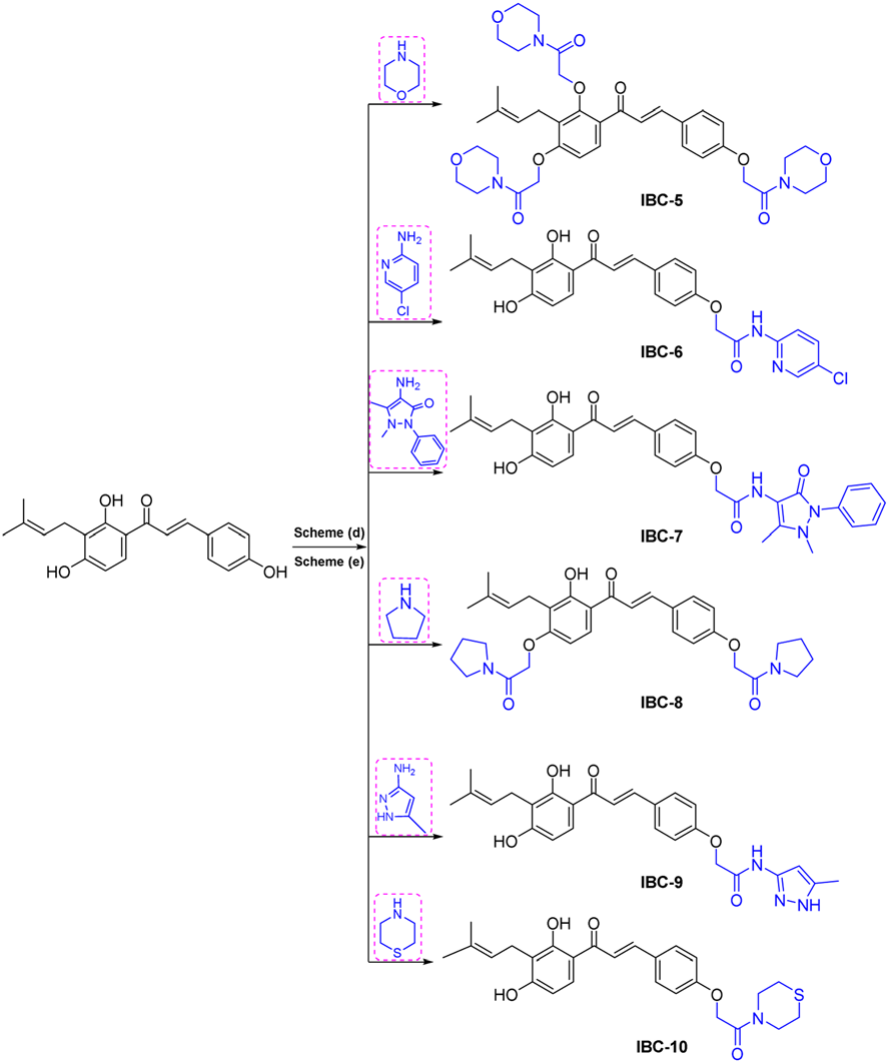
*Reagents and conditions*: scheme (a). Different amine derivatives, Et_3_N, chloroacetylchloride, DCM, 0 °C, 3 h, yield 70–85%; scheme (b). Chloroacetamide derivatives, K_2_CO_3_, DMF, rt, 6 h, product yield 60–75%.

A sufficient amount of IBC was isolated from the seed of *Psoralea corylifolia*. In proton NMR of IBC, the characteristic peaks of α,β-protons of the unsaturated double bond present in the chalcone moiety were observed as doublets at *δ* 7.67 and *δ* 7.51 ppm, whereas the methine (CH) of prenyl chain showed a triplet at *δ* 5.17–5.08 ppm and methylene (CH_2_) was observed at *δ* 3.23 ppm as a doublet signal. Two singlets of methyl groups of the prenyl chain are at *δ* 1.68 and *δ* 1.56 ppm attached to ring A. In the ^13^C NMR of IBC, carbonyl carbon was observed at *δ* 192.30 ppm, whereas α-carbon and β-carbon were observed at *δ* 117.16 and *δ* 143.92 ppm, which confirms that it contains an unsaturated ketone moiety. The carbon of the prenyl chain in IBC was observed for one methylene (CH_2_) at *δ* 21.12 ppm, one methine (CH) at *δ* 117.16 ppm, quaternary carbon at *δ* 130.46 ppm, and two methyl groups at *δ* 24.58 ppm and *δ* 16.54 ppm, respectively. The molecular formula C_20_H_21_O_4_ of IBC was confirmed by HRMS *m*/*z* value 325.1422. This spectroscopic data match their reported data.^13,21^IBC, a prenylated chalcone, was used as a starting compound to generate structurally diverse compounds (IBC-2 to IBC-10) (Scheme 1 and 2). The prenylated chalcone IBC underwent a cyclization reaction to form compound IBC-2 (Scheme 1a). In proton NMR, we have observed two double doublets of two protons of CH_2_ (C-3) at *δ* 3.00 ppm and *δ* 2.80 ppm, and one triplet at *δ* 5.44–5.42 ppm of a proton of CH (C-2). We have observed the cyclization of the α, β-unsaturated ketone moiety, and the cyclization of the prenyl chain, which was confirmed with the ^13^C NMR spectrum. Two CH_2_ groups of prenyl cyclization were observed at *δ* 16.83 and *δ* 31.75 ppm, and one CH_2_ (C-3) was observed at *δ* 44.03 ppm. DEPT-135 has also confirmed all three CH_2_ groups. Oxygenated carbon C-2 (CH) was observed at *δ* 79.29 ppm, and quaternary carbon of the cyclized prenyl chain was observed at *δ* 75.58 ppm, which confirms the structure of IBC-2. HRMS confirmed the molecular formula of C_20_H_21_O_4_ with *m*/*z* 325.1461.

In Scheme 1b, we have carried out a reaction of IBC with mercury(ii) acetate in the presence of pyridine to obtain IBC-3. In ^1^H NMR, we have observed a singlet of a proton at *δ* 6.77 ppm of H-2a and a triplet at *δ* 5.35–5.39 ppm of CH of the prenyl chain, as well as a doublet of two protons of CH_2_ of the prenyl chain at *δ* 3.53 ppm. We also found two methyl singlets at *δ* 1.90 and *δ* 1.74 ppm. In ^13^C NMR, we have observed a carbonyl peak at *δ* 187.64 ppm and a carbon peak at *δ* 150.48 ppm of olefinic C-2. The carbon peak of C-2a was observed at *δ* 115.84 ppm, which was the confirmation peak of the aurone ring with a (*Z*)-isomer.^22^ Carbon peaks of CH_2_ in the prenyl chain were observed at *δ* 25.34, CH at *δ* 127.74, and quaternary carbon at *δ* 135.67 ppm, whereas the carbon of methyl appeared at *δ* 20.62 and *δ* 28.48 ppm. IBC-3 was also confirmed by HRMS with a molecular formula of C_20_H_19_O_4_ in HRMS with *m*/*z* 323.1291. The aurone derivative (IBC-3) from IBC was first synthesized and reported in this article, so IBC-3 is a new compound not reported earlier to the best of our knowledge. It has been observed that one geometrical isomer is formed in this reaction. *Z*-isomers are generally favored over *E*-isomers due to their thermodynamic stability. According to the reported literature, *Z*-isomers had lower potential energies than *E*-isomers, indicating that the *Z*-isomer is preferable, which was also observed in this case.^23,24^ Simple methylation of IBC gave the methylated product IBC-4.

NMR spectrum showed that three hydroxy groups are protected to provide methoxy IBC-4, as confirmed by proton NMR, which showed three singlet peaks of methoxy at *δ* 3.72, *δ* 3.87, and *δ* 3.91 ppm. In ^13^C NMR, peaks of methoxy carbon present at *δ* 55.40, *δ* 55.84, and *δ* 63.06 ppm and molecular formula C_23_H_27_O_4_ were confirmed by HRMS with *m*/*z* 367.1910. IBC-5, IBC-6, IBC-7, IBC-8, IBC-9, and IBC-10 were synthesized by preparing chloroacetamide and reacting with IBC to obtain diverse IBC acetamide derivatives. The confirmation peaks of chalcone moiety (α, β-unsaturated ketone) and carbonyl carbon was found in the range of *δ* 192.35–186.63 ppm, α-carbon was shown in the range of *δ* 118.49–122.51 ppm, and β-carbon was shown in the range of *δ* 142.11–144.90 ppm, which confirms that chalcone moiety is present. The prenyl moiety containing two methyls between *δ* 1–2 ppm and one CH_2_ peak at *δ* 3.3–3.6 ppm and a triplet of one CH peak at *δ* 5.1–5.8 ppm were observed. For IBC-5, we have observed that all three hydroxy groups of IBC were substituted with chloroacetamide of morpholine, which was confirmed with proton NMR, in which 24 protons of twelve CH_2_ of the morpholine peak at *δ* 3.55–3.67 ppm and three CH_2_ of the acetamide group were observed at *δ* 4.51, *δ* 4.74, and *δ* 4.78 ppm. In carbon NMR, three carbonyls of the acetamide group were observed at *δ* 166.09, 166.04, and 165.92 ppm, and carbon of CH_2_ was observed at *δ* 68.16, 67.44, and 66.76 ppm. The synthesized compound was further confirmed by HRMS *m*/*z* 706.3328 with the chemical formula C_38_H_48_N_3_O_10_. IBC-6 has an acetamide derivative of 2-amino-5-chloropyridine, and IBC-7 contains a 4-amino-antipyrine acetamide derivative. In IBC-6 and IBC-7, one hydroxy was substituted that was observed in carbon NMR, and only one CH_2_ peak of acetamide was observed at *δ* 66.19 and *δ* 67.00 ppm, respectively. IBC-8 has pyrrolidine acetamide in which two hydroxy groups were substituted, as observed in the proton NMR with two CH_2_ peaks at *δ* 4.68 and *δ* 4.74 ppm, as well as in carbon NMR with CH_2_ peaks at *δ* 68.17 and *δ* 67.87 ppm. IBC-9 has a 3-amino-5-methyl pyrrole acetamide derivative, and IBC-10 has a thiomorpholine acetamide derivative (Scheme 2). All compounds were characterized and confirmed by NMR spectroscopy and HRMS.

### 2.2 Biology section

#### 2.2.1 Determination of minimum inhibitory concentration (MIC) and minimum bactericidal concentration (MBC)

The antimicrobial activity of all compounds was evaluated by determining their Minimum Inhibitory Concentration (MIC) and Minimum Bactericidal Concentration (MBC) by using against Gram +ve *S. aureus* ATCC 29213, *MRSA* ATCC 15187, Gram −ve *P. aeruginosa* ATCC 27853, and *E. coli* ATCC 25922 pathogens. IBC, IBC-2, and IBC-3 demonstrated robust antibacterial efficacy among the tested compounds. The parent natural prenylated chalcone IBC that contains the chalcone moiety (α, β-unsaturated moiety) showed MIC values of 5 μM and 24 μM against *S. aureus* and *P. aeruginosa*. In contrast, compound IBC-2, having a flavanone moiety in its structure, showed the same potential of antibacterial activity compared to the parent IBC, with a 5.0 μM MIC against *S. aureus* and *P. aeruginosa* with MIC values of 24 μM. IBC and IBC-2 have shown very poor activity against *MRSA* and *E. coli* with MICs >100 μM. Compound IBC-3 contains a five-membered aurone moiety that exhibits broad-spectrum antibacterial activity, which is active against Gram +ve *S. aureus*, *MRSA*, and Gram-ve *E. coli*, *P. aeruginosa* pathogens.

**Fig. 2:**
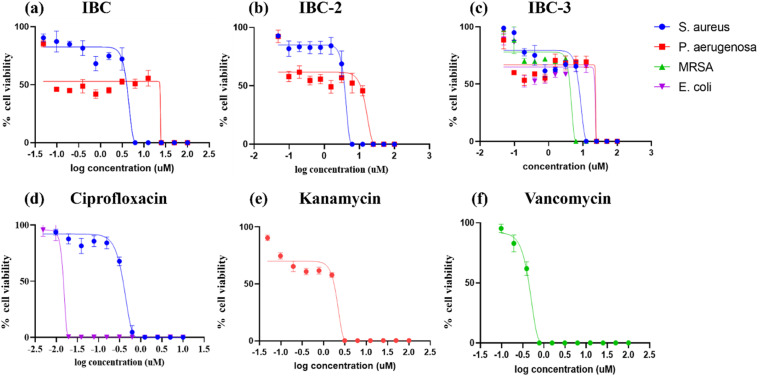
Dose–response curves illustrating the growth inhibition of *S. aureus* ATCC 29213 (blue), *P. aeruginosa* ATCC 27853 (red), *MRSA* ATCC 15187 (green), and *E. coli* ATCC 25922 (purple) by various inhibitors: (a) IBC, (b) IBC-2, and (c) IBC-3. Standard drug controls used against these strains include (d) ciprofloxacin against *S. aureus* (blue) and *E. coli* (purple), (e) vancomycin against *MRSA* (green), and (f) kanamycin against *P. aeruginosa* (red). The compounds (a) IBC and IBC-2 display growth inhibition of Gram-positive (*S. aureus*) and Gram-negative (*E. coli*) bacterial strains, whereas IBC-3 shows antibacterial activity by inhibiting the growth of Gram-positive (*S. aureus* and Methicillin-resistant *S. aureus*) and Gram-negative (*E. coli* and *P. aeruginosa*) bacterial strains. The figure represents the results of three independent experiments.

IBC-3 showed MIC values of 10.0 μM, 6.0 μM, 23.0 μM and 24.0 μM against *S. aureus*, *MRSA*, *E. coli*, and *P. aeruginosa* pathogens, respectively. IBC and IBC-2 displayed antibacterial effects against the Gram-positive strain *S. aureus* but not against the Methicillin-resistant *S. aureus* bacterial strain. *MRSA* is a resistant variant strain of *S. aureus* and possesses intrinsic resistance to methicillin, penicillin, oxacillin, and many antibiotics.^25^ This strain can also acquire resistance to most current antibiotics through mutations or the acquisition of foreign genetic material.^26^ The resistance against *MRSA* being observed against IBC and IBC-2 may be due to either a mutation or their inherent resistance mechanisms. Methylation of IBC (IBC-4) showed decreasing antibacterial activity against all pathogens with MIC value >100 μM. Meanwhile, acetamide derivatives (IBC-5 to IBC-10) showed poor activity against all pathogens. The above results suggest that the cyclization of chalcone (α, β-unsaturated moiety) into a six-membered ring (flavanone moiety) showed no changes in the activity. In contrast, the cyclization of chalcone into a five-membered ring (aurone moiety) showed broad-spectrum antibacterial activity against Gram +ve and Gram −ve pathogens. We have also observed that substituting the hydroxy group of prenylated IBC will decrease the antibacterial activity (IBC-4 to IBC-10). The minimum bactericidal concentration (MBC) is the lowest concentration of an antimicrobial drug that can kill a bacterium within a specified period under controlled conditions. We have also examined the minimum bactericidal concentration (MBC) of the active compounds, *i.e.*, IBC, IBC-2, and IBC-3, which is provided in Table 1. Compound IBC showed MBC values of 6.25 μM and 25 μM, whereas IBC-2 displayed 12.5 μM and 50 μM against *S. aureus* and *P. aeruginosa*, respectively. Compound IBC-3 exhibited MBC values 25 μM, 25 μM, 25 μM, and 100 μM against *S. aureus*, *MRSA*, *E. coli*, and *P. aeruginosa* pathogens, respectively. IBC-3 exhibited a broad-spectrum activity against Gram-negative and Gram-positive strains with significant bactericidal activity. Table 1 displays the minimum inhibitory concentration (MIC) and minimum bactericidal concentration (MBC) of all the compounds against the tested organisms. Fig. 2 illustrates the dose–response curve of these potent inhibitors, IBC, IBC-2, and IBC-3, against *S. aureus*, *P. aeruginosa*, Methicillin-resistant *S. aureus*, and *E. coli* strains (shown in different colors). The standardized positive controls used against these respective strains include ciprofloxacin (*S. aureus* and *E. coli*), vancomycin (*MRSA*), and Kanamycin (*P. aeruginosa*). These curves clearly show a reduction in % age viability with increased concentration of compounds. IBC and IBC-2 exhibited a MIC of 5 μM against *S. aureus* ATCC 29213, whereas the MBC was in the same range as the inhibitory concentration. IBC-3 is bacteriostatic at a lower concentration (25 μM), whereas it is bactericidal at a higher concentration (100 μM).^27^

**Table 1:** MIC and MBC values of compounds against bacterial strains, Gram-positive (*S. aureus* and methicillin-resistant *S. aureus*) and Gram-negative (*E. coli* and *P. aeruginosa*). CC_50_ of compounds against the macrophage cell line RAW 264.7 and selectivity index (SI) of compounds with respect to the bacterial strain *S. aureus*^a^

S. No.	Compounds	Minimum inhibitory concentration (MIC) (μM)				Minimum bactericidal concentration (MBC) (μM)				CC_50_ (μM)	Selectivity index (SI)
		*S. aureus*	*MRSA*	*E. coli*	*P. aeruginosa*	*S. aureus*	*MRSA*	*E. coli*	*P. aeruginosa*		
1	IBC	**5**	>100	>100	**24**	**6.25**	ND	ND	**25**	**94**	**19**
2	IBC-2	**5**	>100	>100	**22**	**12.5**	ND	ND	**50**	**115**	**23**
3	IBC-3	**10**	**6**	**23**	**24**	**25**	**25**	**25**	**100**	**50**	**5**
4	IBC-4	>100	>100	>100	>100	ND	ND	ND	ND	ND	ND
5	IBC-5	>100	>100	>100	>100	ND	ND	ND	ND	ND	ND
6	IBC-6	>100	>100	>100	>100	ND	ND	ND	ND	ND	ND
7	IBC-7	>100	>100	>100	>100	ND	ND	ND	ND	ND	ND
8	IBC-8	>100	>100	>100	>100	ND	ND	ND	ND	ND	ND
9	IBC-9	>100	>100	>100	>100	ND	ND	ND	ND	ND	ND
10	IBC-10	>100	>100	>100	>100	ND	ND	ND	ND	ND	ND
11	Ciprofloxacin	0.62	NA	0.02	NA	ND	ND	ND	ND	ND	ND
12	Vancomycin	NA	0.7	NA	NA	ND	ND	ND	ND	ND	ND
13	Kanamycin	NA	NA	NA	2.8	ND	ND	ND	ND	ND	ND

a NA = not attempted, MIC = minimum inhibitory concentration, MBC = minimum bactericidal concentration, CC_50_ = cytotoxic concentration, SI = selectivity index = CC_50_ of the compound (μM)/MIC of the compound (μM), SI > 10 is considered safe.

#### 2.2.2 Assessment of cell viability of a compound for determining safety indices

**Fig. 3:**
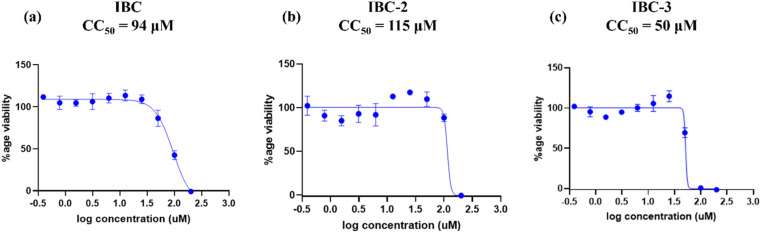
Dose–response curves depict RAW 264.7 cell growth inhibition by (a) IBC, (b) IBC-2, and (c) IBC-3. (a) IBC showing viability of macrophages with CC_50_ 94 μM, (b) IBC-2 showing viability of cells with CC_50_ 115 μM and (c) IBC-3 showing viability of cells with CC_50_ 50 μM. The figure is a representation of three independent experiments.

Given that the antibacterial hits for further investigation must not harm mammalian cell lines and should demonstrate a favorable safety index, the cell viability assay was performed. The safety index is a significant parameter that describes the safety profile of the compound, calculated by dividing the CC_50_ by the MIC of the compound. The high SI gives maximum antibacterial effect with limited cell toxicity. The compounds with SI > 10 are considered safe during *in vitro* assays and approved for further drug development.^28^ The cytotoxic impact of the active compounds was examined using the RAW 264.7 macrophage cell line using the MTT assay. Each compound's CC_50_ (50% cytotoxic concentration) was assessed using different compound concentrations, and the data was analyzed using GraphPad Prism 8.0.2 software. Compound IBC exhibited CC_50_ of 94 μM, and IBC-2 showed CC_50_ of 115 μM, whereas IBC-3 displayed CC_50_ of 50 μM against murine RAW 264.7 macrophage cell line (Fig. 3). The CC_50_ and MIC of each compound against *S. aureus* were employed to calculate the selectivity index for the respective compounds. The selectivity index (SI) concerning *S. aureus* is represented in Table 1. Compound IBC and IBC-2 demonstrated a satisfactory SI (>10), whereas IBC-3 exhibited moderate cytotoxicity with a safety index of less than 10.

#### 2.2.3 Time-kill profiles

**Fig. 4:**
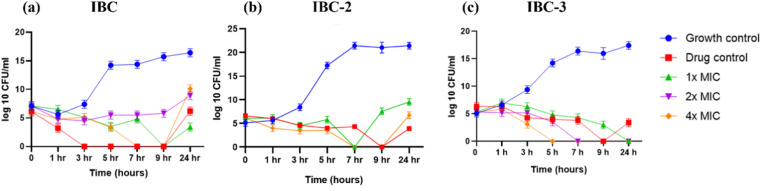
Time-kill curves of (a) IBC, (b) IBC-2, and (c) IBC-3 against *S. aureus*. Ciprofloxacin at 10× MIC was used as drug control. The blue line indicates *S. aureus* growth control, the red line ciprofloxacin drug control, the green line MIC, the purple line 2× MIC, and the orange line 4× MIC of respective compounds. (a) IBC shows concentration-dependent killing till 9 h and becomes resistant after this time-point (b) and (c) compounds IBC-2 and IBC-3 show concentration and time-dependent killing effects. The figure represents the results of three independent experiments.

A time-kill kinetics study was conducted to assess how the activity of compounds changes over time in relation to their concentration against *S. aureus*. Time-kill studies were performed against *S. aureus* ATCC 29213 for IBC, IBC-2, and IBC-3; the results are presented in Fig. 4. For the assay, *S. aureus* was treated with compounds at various concentrations (MIC, 2× MIC, 4× MIC, and 8× MIC) and incubated at 37 °C. Aliquots from the tested samples were taken at different time points (1 h, 3 h, 5 h, 7 h, 9 h, 24 h) and plated on MHA plates. Colony counting was performed following incubation overnight. The colony-forming unit (CFU mL^−1^) was determined, and the time-kill curve was plotted against time. Both IBC-2 and IBC-3 at 2× MIC and 4× MIC demonstrated a decrease in the CFU count of *S. aureus* ATCC 29213 to levels below the detection limit with increasing time. The kill kinetics study showed that IBC-2 and IBC-3 exhibit a concentration-dependent killing effect against *S. aureus* ATCC 29213 over time. However, IBC demonstrates a decrease in CFU with an initial increase in concentration. However, after 24 hours, an increase in CFU was observed, indicating a reversal in the trend of bacterial reduction. This suggests that while the compound exhibits concentration-dependent killing initially, its effectiveness diminishes over time, potentially leading to bacterial regrowth, showing the bacteriostatic nature of the compounds.

#### 2.2.4 Evaluating the synergistic efficacy of test compounds with standard drugs

**Fig. 5:**
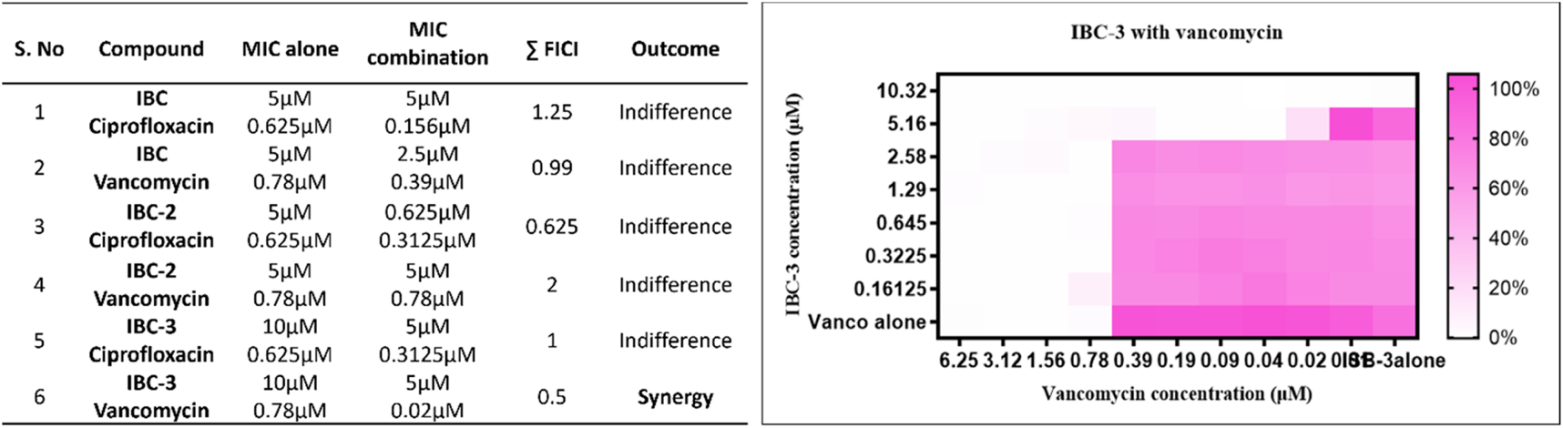
A table on the left shows the combinational effect of compounds IBC, IBC-2, and IBC-3 with standard drugs ciprofloxacin and vancomycin. IBC-3 showed a synergistic effect with vancomycin and a complementary effect. The heatmap on the right side displays the synergistic impact of IBC-3 with vancomycin, analyzed by checkerboard analysis against *S. aureus*. The degree of inhibition is shown as a heat plot where the lightest point represents maximum inhibition, whereas the darkest pink point represents maximum growth of *S. aureus*.

The identified compounds may facilitate the activity of other antibiotics and act synergistically with them. Hence, the checkerboard microdilution method was used to study the effect of the active compounds (IBC, IBC-2, and IBC-3) on the MIC of known antibiotics for *in vitro* combination studies. The MIC of the compounds was determined in the presence of other antibiotics (ciprofloxacin and vancomycin). The Fractional Inhibitory Concentration Index (FICI) was calculated for each test compound and reference drug to find the combinational effect. The computed FICI and results are presented in Table 2. Among the three, IBC-3 demonstrated a synergistic effect with vancomycin against *S. aureus*. The heatmap for the interaction of IBC-3 with vancomycin is shown in Fig. 5. A series of combination studies were conducted to investigate further the potentiating combinations of the active compounds and known antibiotics. Notably, IBC-3 showed a synergistic effect with vancomycin against *S. aureus*. As vancomycin primarily targets the cell wall, IBC-3 might exert its inhibitory effects through a complementary mechanism of action, leading to a synergistic impact when combined, effectively inhibiting bacterial growth.

**Table 2:** *In silico* ADMET properties of compounds IBC, IBC-2, and IBC-3

Parameters	IBC	IBC-2	IBC-3
Molecular formula	C_20_H_20_O_4_	C_20_H_20_O_4_	C_20_H_18_O_4_
Molecular weight	324.37	324.37	322.35
TPSA (Å^2^)	77.76	55.76	66.76
*M* log *P*	2.70	2.46	2.31
*W* log *P*	4.10	3.88	4.11
*X* log *P*3	5.10	3.53	4.81
Fraction C_sp^3^_	0.15	0.35	0.15
Log S (ESOL)	−5.10	−4.38	−5.04
Synthetic accessibility	3.03	3.51	3.37
Rotatable bonds	5	1	3
HBA	4	4	4
HBD	3	1	2
Lipinski violation	0	0	0
Druglikeness-Lipinski (violation)	Yes (0)	Yes (0)	Yes (0)
Druglikeness-Ghose	Yes	Yes	Yes
Druglikeness-Veber	Yes	Yes	Yes
Druglikeness-Egan	Yes	Yes	Yes
Druglikeness-Muegge (violation)	No (1)	Yes	Yes
GI absorption	High	High	High
BBB permeant	No	Yes	Yes
CYP1A2 inhibitor	Yes	Yes	Yes
CYP2C19 inhibitor	No	Yes	Yes
CYP2C9 inhibitor	Yes	Yes	Yes
CYP2D6 inhibitor	No	Yes	No
CYP3A4 inhibitor	Yes	Yes	Yes

#### 2.2.5 IBC-3 disrupts the membrane integrity of *S. aureus* as observed through SEM analysis

IBC is an antibacterial compound with various mechanisms of action related to membrane disruption in bacteria. It acts by membrane damage to Methicillin-Susceptible *Staphylococcus aureus* (MSSA), which inhibits macromolecular production.^29^ After binding to the phospholipids of the MSSA membrane, it produces metabolic disruptions and the dissipation of the proton motive force.^30^ Additionally, it has been reported that IBC perturbs the membranes of human colorectal cancer cells and can intercalate into model membranes, influencing the phase transition of phospholipids.^31^ The antibacterial activity of isobavachalcone (IBC) has been linked to membrane disruption, according to a mechanism published by Leticia Ribeiro de Assis and co-authors.^17^

**Fig. 6:**
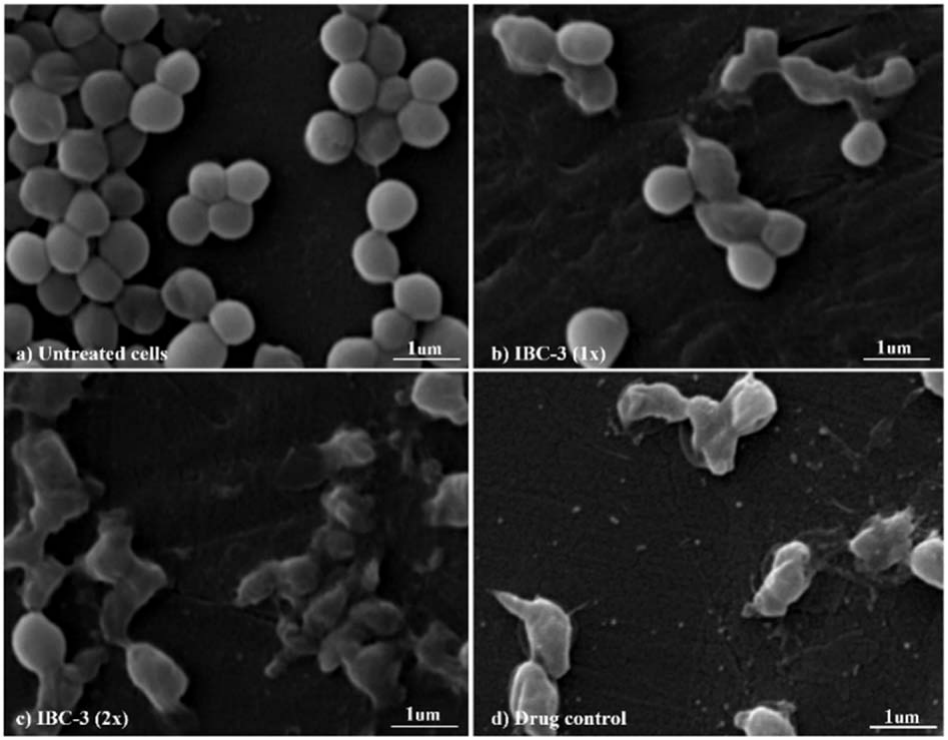
Scanning electron micrographs revealing the morphological changes in *S. aureus* concerning different circumstances. (a) Untreated cells, (b) cells treated with compound IBC-3 at 1× MIC, (c) cells treated with IBC-3 at 2× MIC, (d) drug control, vancomycin at 4× MIC. The images display the complete disruption in cell integrity in IBC-3 treated cells at both concentrations, 1× MIC and 2× MIC, similar to drug control by vancomycin.

IBC-3, a derivative of IBC, showed potency with broad-spectrum antimicrobial activities against Gram +ve (*S. aureus* and *MRSA*) and Gram −ve (*E. coli* and *P. aeruginosa*) strains. So, we have taken IBC-3 for observing morphological changes in *S. aureus*. The scanning electron micrographs display the alterations in the morphological appearance of bacterial cells when treated with IBC-3, similar to the standard drug vancomycin. Fig. 6 shows bacterial cells with bulging surfaces at 1× MIC and complete disruption of cell integrity at 2× MIC of the test compound, respectively. The drug-control vancomycin shows complete disruption of bacterial cells, while the untreated cells are beautifully arranged in bunches with smooth surfaces and spherical shapes. These findings suggest that IBC-3 might target the cytoplasmic membrane or cell wall of *S. aureus*.

#### 2.2.6 IBC-3 disrupts cell integrity as confirmed by PI staining

**Fig. 7:**
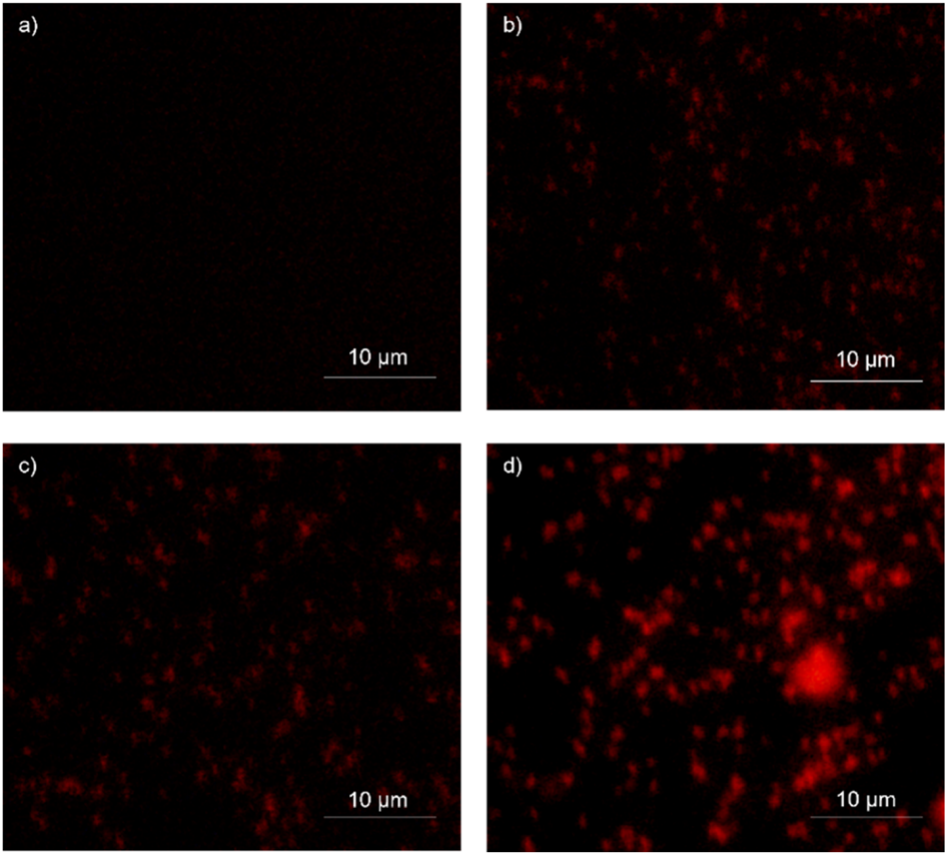
Fluorescence images of PI-stained *S. aureus* under different conditions: (a) untreated cells, (b) vancomycin-treated cells at 1× MIC, (c) IBC-3-treated cells at 1× MIC, and (d) IBC-3-treated cells at 2× MIC. The images reveal enhanced red fluorescence emission in IBC-3-treated cells at both concentrations, similar to the effect seen in the vancomycin-treated cells. Untreated cells do not exhibit red fluorescence, as expected. These results demonstrate the significant cell permeability of IBC-3 against *S. aureus*.

To further validate the effect of IBC-3 on *S. aureus* bacterial cells, we conducted PI (propidium iodide) staining. PI is a nucleic acid-binding fluorophore that emits a red fluorescence signal. It is generally impervious to viable bacterial cells. Still, it can easily permeate through compromised and dead cells and bind to nucleic acid, giving red fluorescent signals.^32^ To assess the impact of IBC-3 on *S. aureus* cell integrity, various concentrations (1× MIC and 2× MIC) of the compound were tested. Vancomycin used as a drug control was applied at a concentration of 1× MIC. The untreated *S. aureus* bacterial cells were used as growth control, where a minimum number of dead cells was expected. Significantly, a higher number of dead cells emitting red fluorescence were observed in the vancomycin-treated sample, indicating its potent effect on the cell wall. Similarly, treatment with IBC-3 resulted in a notably increased intensity of red fluorescence compared to the untreated cells, suggesting its disruptive action on cell integrity, as depicted in the figure (Fig. 7).

### 2.3 *In silico* ADMET study

**Fig. 8:**
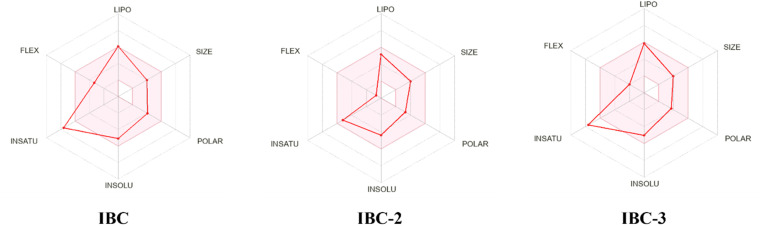
The bioavailability radar depicts the drug-likeness of a compound. The ink zone in the bioavailability radar chart represents the optimum range for each parameter: flexibility (up to 9 rotational bonds), lipophilicity (XLOGP3 between −0.7 to +5.0), size (molecular weight 150–500 g mol^−1^), polarity (TPSA 20–130 Å^2^), solubility: (log *S* ≤ 6), saturation (carbon fraction in the sp^3^ hybridization ≥ 0.25). Compound IBC exhibits increased saturation, while IBC-2 falls within all specified ranges. Conversely, IBC-3 shows higher saturation levels. Thus, IBC-2 demonstrates more potential for oral bioavailability than IBC and IBC-3.

During drug development, there is a critical need to identify the medicinal properties of molecules to confirm the therapeutic significance of the target compounds, drug-likeness properties or physicochemical properties, and pharmacokinetic properties.^33^ Additionally, the Lipinski rule of five (RO5), which relies on molecular characteristics such as the molecular weight, count of hydrogen bond acceptors (H-ba), count of hydrogen bond donors (H-bd), and cLogP (partition coefficient between *n*-octanol and water), have been extensively employed as a criterion in the selection process for active drug molecules. Lipinski's rule of five serves as the gold standard for assessing the potential oral bioavailability of a compound or drug. Compounds that do not meet more than one criterion of this rule are unlikely to succeed in drug development due to challenges related to permeability or solubility.^34^ The *in silico* study supports the findings of the *in vitro* study and contributes to the conclusion that the IBC, IBC-2, and IBC-3 exhibit more effective drug-like properties for antibacterial activity (Fig. 8).

*In silico* prediction for furthering their potential to act as drug molecules based on their physicochemical and ADMET properties of IBC, IBC-2, and IBC-3 were performed using SwissADME software, as represented in Table 2. An online tool was employed to establish correlations between the chemical structure, ADME (absorption, distribution, metabolism, and excretion) properties, and compound efficacy. The prediction results revealed that IBC-3 possesses several favorable pharmacokinetic properties compared to IBC. Both IBC-2 and IBC-3 conform to all the criteria for drug-likeness, including Lipinski's rule, Ghose's rule, Veber's rule, Egan's rule, and Muegge's rule, whereas IBC violates Drug likeness-Muegge (violation) rule. IBC-2 and IBC-3 showed variation in ADME properties compared to IBC, like BBB permeation. IBC-2 showed one H-bond donor, while IBC-3 displayed two H-bond donors. The molecular weight of active compounds is less than 500 g mol^−1^. Log *P* (*X* log *P*) analyzed the lipophilicity of compounds, showing a favorable range of 2.31–5.1.

**Fig. 9:**
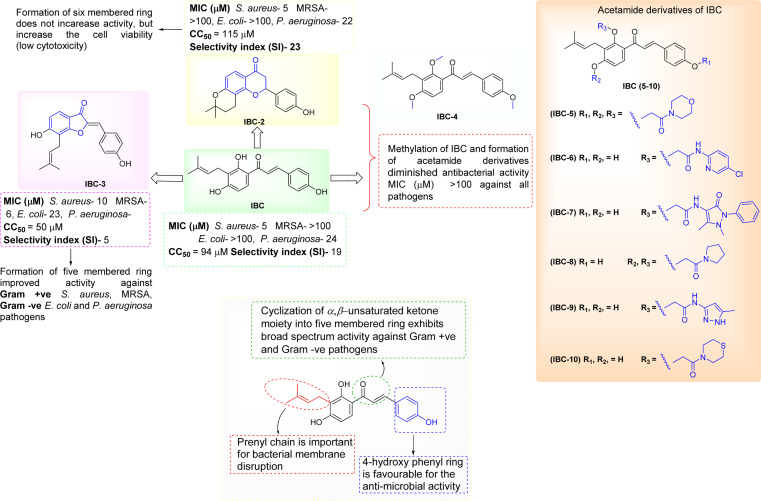
Structure–activity relationships of structurally diverse IBC derivatives.

The synthesized diverse derivatives (IBC-2 to IBC-10) of natural prenylated IBC were tested for antibacterial activity against Gram +ve *S. aureus*, *MRSA*, and Gram −ve *E. coli*, *P. aeruginosa* pathogens. Several structural features influence the structure–activity relationship (SAR) of prenylated natural products, like its core structure, the prenyl group, and functional groups. IBC contains the chalcone scaffold (α, β-unsaturated moiety) with a prenyl chain in the A-ring, which showed activity against *S. aureus* and *P. aeruginosa*. IBC-2 contains a six-membered flavanone moiety and also showed no significant improvement in the activity, whereas IBC-3 contains a five-membered aurone moiety, which displayed activity against Gram +ve *S. aureus*, *MRSA*, and Gram −ve *E. coli*, *P. aeruginosa* pathogens. That suggests that chalcone moiety's cyclization into a five-membered ring displayed broad-spectrum activity (Fig. 9).

In this work, we observed that where hydroxy groups of natural flavonoid IBC were substituted, methylation (IBC-4) and acetamide derivatives (IBC-5 to IBC-10) did not significantly contribute to antibacterial properties when tested to assess their *in vitro* antimicrobial activity. For instance, the methylated product and acetamide derivatives of IBC did not show any potency even though the prenyl moiety is attached to the backbone and at the position of the phenolic skeleton, which was a prerequisite for antibacterial activities, as previously reported, wherein the prenylation at appropriate positions plays a critical role in the antibacterial activity, particularly MDR pathogens.^35^ We observed that IBC-3, which is an aurone derivative, exhibited a broad-spectrum activity against Gram-negative and Gram-positive strains, unlike IBC and IBC-2, which were active only in Gram-positive strains (Fig. 9). IBC has been reported to be involved in drug–drug interactions^36^ and is subject to export through efflux pump mechanisms.^37^ However, due to their low toxicity, their development as a potential antibacterial agent is considered an advantage.^38^ Further, some reports suggest that the prenylation increased the lipophilicity of flavonoids, which propagates to an increase in affinity to the biological membranes and improved interactions with target proteins.^35,39^IBC and chalcone scaffolds are associated with membrane disruption, which is a promising target for screening antibiotic candidates and has potential applications as an antibacterial drug and a medical device coating.^17,40^ Our study suggests that IBC-3 might target the cell wall of *S. aureus* as the cell integrity is disrupted compared with similar standard drug vancomycin (Fig. 6). Observation of this antibacterial structure–activity relationship of these diverse IBC derivatives and their structural features have similar inference with the study of prenylflavaone derivatives reported earlier.^41^

Most currently used antibiotics target Gram-positive bacteria, while Gram-negative bacteria easily bypass the action of most drug molecules because of their unique outer membrane, which acts as a potent barrier restricting the entry of compounds into the cell.^42^ To increase the drug penetration of the compounds into Gram-negative bacteria, the physicochemical properties can help to accumulate, target, or bypass the outer-mediated barrier in Gram-negative bacterial pathogens.^43^IBC-3 has favorable physiochemical properties that differ from IBC and IBC-2, as evident in our *in silico* studies, which could be another reason IBC-3 shows activity against both Gram-positive and Gram-negative bacteria. Furthermore, IBC contains the chalcone moiety with a prenyl chain that is reported to show antibacterial activity. IBC is a starting natural compound for synthesizing structurally diverse derivatives (IBC-2 and IBC-3). After cyclization reactions, IBC-2 and IBC-3 were obtained. IBC-2 contains the six-membered cyclized ring (flavanone moiety) in its structure, whereas IBC-3 contains the five-membered cyclized ring (aurone ring). According to the literature, aurones have promising antibacterial activity against Gram-positive (*MRSA*) and Gram-negative bacteria. This might be possible due to the structural feature changes in the compounds.^20,44^

## 3. Materials and methods

### 3.1 Extraction and isolation procedure of IBC from *Psoralea corylifolia*

*Psoralea corylifolia* L. seeds were collected and authenticated from the CSIR-IIIM herbarium. The dried plant seed was pulverized, and sequential extraction was carried out using hexane, dichloromethane, and methanol as extraction solvents. 500.0 gm of *P. corylifolia* L. seeds were extracted using 2.5 L of DCM, kept overnight, filtered out, and evaporated using a rota-vapor. This procedure was repeated three times. 32.0 g of DCM extract was obtained after evaporating the solvent. This extract was loaded in column chromatography using silica 60–120 mesh and run the column in ethyl acetate : hexane, increasing its polarity to 10% ethyl acetate : hexane. We have used the DCM extract, though the original isolation procedure reported by Suk Dev and co-workers used chloroform extract for the petroleum ether, exhausting the whole seeds of the plant.^13^ Here, isobavachalcone was isolated from the dichloromethane extract using column chromatography to obtain a sufficient amount, and its purity was checked with HPLC, which gives up to 98% percentage purity (see ESI†).

### 3.2 Synthesis of IBC-2

50.0 mg of IBC was taken in a round bottom flask and dissolved in methanol and water (1 : 1 ratio). Then, 10% aqueous NaOH was added dropwise. The reaction mixture was stirred for 4 hours at room temperature. After completion of the reaction, 50% aqueous H_2_SO_4_ was added to the reaction mixture dropwise, resulting in a yellow precipitate that was filtered out and purified using silica gel column chromatography in the mobile phase of 30% ethyl acetate in hexane using the reported procedure with slight modifications.^45^

### 3.3 Synthesis ofIBC-3

IBC (50 mg) was taken in RBF and dissolved in pyridine (5 mL), then added to Hg(OAc)_2_ (1 eq.). This reaction was refluxed at 110 °C for 5 hours. The reaction was monitored using thin-layer chromatography. After completion of the reaction, the solvent was evaporated using rota vapor, and the work-up was done using ethyl acetate and water. The organic layer was collected and evaporated. The pure compound was purified by column chromatography in the mobile phase of 40% ethyl acetate in hexane.^46^

### 3.4 Methylation of IBC (IBC-4)

IBC (50 mg) was dissolved in DMF (3 mL) and methyl iodide (1.2 eq.), then slowly NaH (1.2 eq.) at 0 °C was added. The reaction was kept for 3 hours at room temperature. After checking the TLC, the work-up was done. The pure compound was isolated using column chromatography in the mobile phase of 20% ethyl acetate in hexane.^47^

### 3.5 Acetamide derivatives of IBC (IBC-5–10)

Different nitrogen-containing amino compounds were dissolved in HPLC grade DCM, and then triethylamine was added in a round bottom flask. Chloroacetyl chloride was added dropwise into the reaction mixture at 0 °C. The resulting mixture was stirred for 3 h at room temperature. After observing the reaction in TLC, the solvent was evaporated on rotavapor, and then a work-up was done with an organic solvent (ethyl acetate) and water. The ethyl acetate layer was collected and dried with Na_2_SO_4_, and the solvent was evaporated using a rotavapor; this gives chloro-containing derivatives of different moieties of amines. The chloro derivative reacted with isobavachalcone in DMF, and K_2_CO_3_ was added to the reaction mixture. The reaction was stirred at room temperature for 6 hours. The reaction was checked in TLC after the reaction work-up was completed. The pure compound was obtained using silica column chromatography.^48,49^

IBC: ^1^H NMR (400 MHz, MeOD) *δ* 7.72 (d, *J* = 9.0 Hz, 1H), 7.67 (d, *J* = 15.4 Hz, 1H), 7.51 (dd, *J* = 6.8, 4.9 Hz, 3H), 6.77–6.71 (m, 2H), 6.33 (d, *J* = 8.9 Hz, 1H), 5.17–5.08 (m, 1H), 3.26–3.18 (m, 3H), 1.68 (d, *J* = 0.7 Hz, 3H), 1.56 (d, *J* = 0.9 Hz, 3H).; ^13^C NMR (101 MHz, MeOD) *δ* 192.30, 163.76, 162.24, 160.07, 143.92, 130.46, 130.36, 129.02, 126.52, 122.24, 117.16, 115.51, 115.17, 113.14, 106.82, 24.58, 21.12, 16.54.; HRMS (ESI) calcd for C_20_H_20_O_4_; 325.1440 [M + H]^+^; found 325.1422.

#### 3.5.1 IBC-2: 2-(4-hydroxyphenyl)-8,8-dimethyl-2,3,9,10-tetrahydro-4*H*,8*H*-pyrano[2,3-*f*]chromen-4-one

Yield −60%, ^1^H NMR (400 MHz, CDCl_3_) *δ* 7.72 (d, *J* = 8.8 Hz, 1H), 7.36 (d, *J* = 8.4 Hz, 2H), 6.93–6.87 (m, 2H), 6.50 (d, *J* = 8.8 Hz, 1H), 5.45–5.42 (t, 1H), 3.00 (dd, *J* = 16.8, 13.3 Hz, 1H), 2.80 (dd, *J* = 16.8, 3.0 Hz, 1H), 2.68 (dd, *J* = 12.2, 6.5 Hz, 2H), 1.79 (dd, *J* = 6.7, 3.7 Hz, 2H), 1.36 (s, 3H), 1.34 (s, 3H).; ^13^C NMR (101 MHz, CDCl_3_) *δ* 191.41, 160.89, 160.80, 155.97, 131.38, 127.72, 125.84, 115.58, 113.63, 112.02, 109.15, 79.29, 75.58, 44.03, 31.75, 27.05, 26.39, 16.83.; HRMS (ESI) calcd for C_20_H_20_O_4_; 325.1440 [M + H]^+^; found 325.1461.

#### 3.5.2 IBC-3: (*Z*)-6-hydroxy-2-(4-hydroxybenzylidene)-7-(3-methylbut-2-en-1-yl)benzofuran-3(2*H*)-one

Yield −70%, ^1^H NMR (400 MHz, MeOD) *δ* 7.85 (d, *J* = 8.7 Hz, 2H), 7.48 (d, *J* = 8.4 Hz, 1H), 6.89 (d, *J* = 8.7 Hz, 2H), 6.77 (s, 1H), 6.72 (d, *J* = 8.4 Hz, 1H), 5.37 (t, *J* = 7.2 Hz, 1H), 3.53 (d, *J* = 7.2 Hz, 2H), 1.90 (s, 3H), 1.74 (s, 3H); ^13^C NMR (101 MHz, CDCl_3_) *δ* 187.64, 170.21, 167.93, 163.43, 150.48, 137.18, 135.67, 127.74, 126.52, 125.03, 119.49, 117.32, 116.44, 115.84, 28.48, 25.34, 20.62.; HRMS (ESI) calcd for C_20_H_19_O_4_; 323.1291 [M + H]^+^; found 323.1291.

#### 3.5.3 IBC-4: (*E*)-1-(2,4-dimethoxy-3-(3-methylbut-2-en-1-yl) phenyl)-3-(4-methoxyphenyl)prop-2-en-1-one

Yield −75%, ^1^H NMR (400 MHz, CDCl_3_) *δ* 7.71 (dd, *J* = 17.9, 7.7 Hz, 1H), 7.65–7.56 (m, 3H), 7.46–7.40 (m, 1H), 6.95 (dd, *J* = 9.1, 6.0 Hz, 2H), 6.73 (dd, *J* = 17.6, 6.5 Hz, 1H), 5.24–5.17 (m, 1H), 3.92–3.88 (m, 3H), 3.88–3.82 (m, 3H), 3.72 (s, 3H), 3.41 (t, *J* = 8.7 Hz, 2H), 1.82 (s, 3H), 1.72 (s, 3H).; ^13^C NMR (101 MHz, CDCl_3_) *δ* 191.78, 161.43, 158.87, 143.10, 131.62, 130.17, 129.57, 127.94, 126.34, 124.12, 123.91, 122.70, 114.36, 106.35, 63.06, 55.84, 55.40, 25.78, 22.80, 17.87.; HRMS (ESI) calcd for C_23_H_27_O_4_; 367.1909 [M + H]^+^; found 367.1910.

#### 3.5.4 IBC-5: (*E*)-2,2′-((2-(3-methylbut-2-en-1-yl)-4-(3-(4-(2-morpholino-2-oxoethoxy)phenyl) acryloyl)-1,3-phenylene) bis(oxy)) bis (1-morpholinoethan-1-one)

Yield −65%, mobile phase for purification (30% ethyl acetate : hexane) using silica column chromatography. ^1^H NMR (400 MHz, CDCl_3_) *δ* 7.59 (t, *J* = 12.8 Hz, 3H), 7.53 (d, *J* = 8.7 Hz, 1H), 7.34–7.26 (m, 1H), 6.97 (d, *J* = 8.7 Hz, 2H), 6.79 (d, *J* = 8.7 Hz, 1H), 5.13 (t, *J* = 6.6 Hz, 1H), 4.78 (s, 2H), 4.74 (s, 2H), 4.51 (s, 2H), 3.72–3.50 (m, 24H), 3.48 (d, *J* = 6.5 Hz, 2H), 1.75 (s, 3H), 1.68 (s, 3H); ^13^C NMR (101 MHz, CDCl_3_) *δ* 191.60, 166.09, 166.04, 165.92, 159.72, 159.40, 156.91, 144.16, 132.14, 130.50, 129.43, 128.45, 126.93, 124.55, 124.32, 122.51, 115.04, 107.57, 73.44, 68.16, 67.44, 66.76, 66.67, 66.51, 45.86, 45.35, 42.45, 42.02, 25.79, 23.07, 18.01.; HRMS (ESI) calcd for C_38_H_48_N_3_O_10_; 706.3340 [M + H]^+^; found 706.3328.

#### 3.5.5 IBC-6: (*E*)-*N*-(5-chloropyridin-2-yl)-2-(4-(3-(2,4-dihydroxy-3-(3-methylbut-2-en-1-yl) phenyl)-3-oxoprop-1-en-1-yl) phenoxy) acetamide

Yield −70%, mobile phase for purification (30% ethyl acetate: hexane) using silica column chromatography. ^1^H NMR (400 MHz, MeOD) *δ* 8.30–8.19 (m, 2H), 7.91–7.66 (m, 4H), 7.59–7.45 (m, 2H), 7.09 (d, *J* = 8.8 Hz, 1H), 6.89 (d, *J* = 8.6 Hz, 1H), 6.50 (dd, *J* = 35.8, 8.9 Hz, 1H), 5.26 (d, *J* = 7.1 Hz, 1H), 4.76 (d, *J* = 21.3 Hz, 2H), 3.37 (d, *J* = 6.5 Hz, 2H), 1.82 (s, 3H), 1.70 (s, 3H).; ^13^C NMR (101 MHz, MeOD) *δ* 191.25, 163.02, 161.65, 159.31, 158.19, 145.66, 145.58, 142.11, 137.43, 129.88, 129.50, 128.28, 121.30, 120.90, 118.49, 116.06, 115.06, 114.32, 112.47, 106.48, 102.05, 66.19, 24.51, 20.66, 16.53; HRMS (ESI) calcd for C_27_H_26_N_2_O_5_Cl; 493.1530 [M + H]^+^; found 493.1529.

#### 3.5.6 IBC-7: (*E*)-2-(4-(3-(2,4-dihydroxy-3-(3-methylbut-2-en-1-yl) phenyl)-3-oxoprop-1-en-1-yl) phenoxy)-*N*-(1,5-dimethyl-3-oxo-2-phenyl-2,3-dihydro-1*H*-pyrazol-4-yl) acetamide

Yield −62%, mobile phase for purification (40% ethyl acetate : hexane) using silica column chromatography. ^1^H NMR (400 MHz, CDCl_3_) *δ* 13.78 (s, 1H), 8.86 (s, 1H), 8.44 (s, 1H), 7.73 (d, *J* = 15.4 Hz, 1H), 7.52 (d, *J* = 8.1 Hz, 5H), 7.42–7.38 (m, 4H), 6.89 (d, *J* = 8.7 Hz, 2H), 6.49 (d, *J* = 8.9 Hz, 1H), 5.32 (t, *J* = 7.2 Hz, 1H), 4.55 (s, 2H), 4.19 (s, 2H), 3.20 (s, 3H), 2.31 (s, 3H), 1.83 (s, 3H), 1.75 (s, 3H).; ^13^C NMR (101 MHz, CDCl_3_) *δ* 191.57, 167.48, 166.01, 164.06, 162.01, 158.80, 143.01, 133.37, 130.26, 129.61, 129.57, 128.93, 128.32, 125.59, 125.44, 121.84, 118.75, 115.07, 113.59, 106.44, 67.00, 42.61, 35.32, 25.82, 17.95, 11.97.; HRMS (ESI) calcd for C_33_H_34_N_3_O_6_; 568.2448 [M + H]^+^; found 568.2457.

#### 3.5.7 IBC-8: (*E*)-1-(2-hydroxy-3-(3-methylbut-2-en-1-yl)-4-(2-oxo-2-(pyrrolidin-1-yl) ethoxy) phenyl)-3-(4-(2-oxo-2-(pyrrolidin-1-yl) ethoxy) phenyl) prop-2-en-1-one

Yield −73%, mobile phase for purification (25% ethyl acetate : hexane) using silica column chromatography. ^1^H NMR (400 MHz, CDCl_3_) *δ* 13.45 (s, 1H), 7.87–7.75 (m, 2H), 7.60 (d, *J* = 8.8 Hz, 2H), 7.50–7.43 (m, 1H), 7.02–6.94 (m, 2H), 6.52 (dd, *J* = 8.9, 6.0 Hz, 1H), 5.22–5.21 (t, 1H), 4.74 (s, 2H), 4.68 (s, 2H), 3.55–3.50 (m, 8H), 3.43 (d, *J* = 7.0 Hz, 2H), 2.02–1.92 (m, 4H), 1.86 (m, *J* = 13.6, 6.5 Hz, 4H), 1.79 (s, 3H), 1.68 (s, 3H); ^13^C NMR (101 MHz, CDCl_3_) *δ* 192.35, 166.15, 165.95, 163.21, 161.51, 160.16, 144.00, 131.83, 130.36, 129.15, 128.40, 127.58, 122.17, 120.99, 118.51, 117.86, 115.17, 102.95, 68.71, 67.87, 46.26, 46.14, 46.01, 26.27, 25.78, 23.80, 21.88, 17.87; HRMS (ESI) calcd for C_32_H_39_N_2_O_6_; 547.2808 [M + H]^+^; found 547.2806.

#### 3.5.8 IBC-9: (*E*)-2-(4-(3-(2,4-dihydroxy-3-(3-methylbut-2-en-1-yl)phenyl)-3-oxoprop-1-en-1-yl)phenoxy)-*N*-(5-methyl-1*H*-pyrazol-3-yl)acetamide

Yield −70%, mobile phase for purification (35% ethyl acetate : hexane) using silica column chromatography. ^1^H NMR (400 MHz, MeOD) *δ* 7.77–7.61 (m, 4H), 7.60–7.51 (m, 1H), 7.00 (d, *J* = 8.8 Hz, 2H), 6.34 (d, *J* = 8.9 Hz, 1H), 6.25 (s, 1H), 5.13 (t, *J* = 7.2 Hz, 1H), 4.63 (s, 2H), 3.22 (d, *J* = 5.2 Hz, 2H), 2.18 (s, 3H), 1.68 (s, 2H), 1.56 (s, 3H); ^13^C NMR (101 MHz, MeOD) *δ* 186.63, 162.73, 161.27, 159.38, 157.42, 153.91, 144.90, 138.06, 125.44, 124.80, 124.72, 124.16, 123.15, 120.34, 117.35, 113.87, 110.20, 108.65, 102.77, 101.08, 62.15, 37.86, 21.08, 13.21, 7.26.; HRMS (ESI) calcd for C_26_H_28_N_3_O_5_; 462.2029 [M + H]^+^; found 462.2031.

#### 3.5.9 IBC-10: (*E*)-1-(2,4-dihydroxy-3-(3-methylbut-2-en-1-yl)phenyl)-3-(4-(2-oxo-2-thiomorpholinoethoxy) phenyl)-prop-2-en-1-one

Yield −72%, mobile phase for purification (45% ethyl acetate : hexane) using silica column chromatography. ^1^H NMR (400 MHz, Pyr) *δ* 7.77 (d, *J* = 15.3 Hz, 1H), 7.69 (d, *J* = 8.9 Hz, 1H), 7.55 (d, *J* = 15.4 Hz, 1H), 7.40 (d, *J* = 8.7 Hz, 2H), 6.87 (s, 1H), 6.85–6.84 (m, 1H), 6.50 (d, *J* = 8.8 Hz, 1H), 5.39 (t, *J* = 7.2 Hz, 1H), 4.70 (s, 2H), 3.54 (s, 2H), 3.49 (d, *J* = 7.2 Hz, 2H), 3.39 (s, 2H), 2.29 (s, 2H), 2.17 (s, 2H), 1.56 (s, 3H), 1.33 (s, 3H).; ^13^C NMR (101 MHz, Pyr) *δ* 192.18, 166.02, 164.93, 163.89, 160.62, 143.61, 131.04, 130.73, 129.88, 128.61, 123.88, 119.27, 116.07, 115.51, 113.67, 107.92, 67.18, 47.80, 44.49, 29.75, 27.17, 25.69, 22.32, 17.85.; HRMS (ESI) calcd for C_26_H_30_NO_5_S; 468.1845 [M + H]^+^; found 468.1838.

### 3.6 Bacterial strains and culture conditions

The bacterial strains *S. aureus* ATCC 29213, *MRSA* ATCC 15187, *E. coli* ATCC 25922, and *P. aeruginosa* ATCC 27853 were obtained from ATCC (American Type Culture Collection). Cation-adjusted Mueller–Hinton broth (MHB; Becton–Dickinson, Cockeysville, MD) was used to determine the minimum inhibitory concentration (MIC). Mueller–Hinton agar (MHA; Becton–Dickinson) was used for minimum bactericidal concentration (MBC) studies. Alamar blue (Himedia) was used to detect the MIC value.

RAW 264.7 cells were acquired from the American type culture collection (ATCC, Manassas, VA) and cultured in Dulbecco's modified eagle's medium (DMEM, Gibco, Life Technologies, NY) supplemented with 10% fetal bovine serum (FBS) at 37 °C and 5% CO_2_. MTT dye (Himedia) was used to detect CC_50_ values. The antibiotics ciprofloxacin hydrochloride monohydrate (Himedia), kanamycin sulfate (Himedia), and vancomycin (Himedia) were used for reference in microbiological assays.

### 3.7 MIC (minimum inhibitory concentration) and MBC (minimum bactericidal concentration) determination

The antibacterial activity of the compounds against *S. aureus* ATCC 29213, *MRSA* ATCC 15187, *E. coli* ATCC 25922, and *P. aeruginosa* ATCC 27853 was determined through a broth microdilution method according to the guidelines of the Clinical and Laboratory Standards Institute (CLSI).^50^ For this experiment, the compounds and selective antibiotics (Ciprofloxacin for *S. aureus* and *E. coli*, Vancomycin for *MRSA*, and Kanamycin for *P. aeruginosa*) were dissolved in DMSO and tested against both Gram-positive and Gram-negative bacterial strains. Growth control and media control were employed along with 2% DMSO as vehicle control. Standardized positive controls used against these strains included ciprofloxacin (*S. aureus* and *E. coli*), vancomycin (*MRSA*), and kanamycin (*P. aeruginosa*).

In a 96-well flat bottom plate, the test compounds were two-fold serially diluted in Mueller Hinton Broth (MHB). The final concentrations of the compounds ranged from 100 to 0.08 μM. 100 μL of standardized bacterial inoculum with turbidity equivalent to 0.5 McFarland standard (1.5 × 108 CFU mL^−1^) was diluted 1 : 100 (1.5 × 10^6^ CFU mL^−1^) in Mueller Hinton Broth and inoculated in the plates.^51^ The plates were incubated for 24 hours at 37 °C. After incubation, 10 μL of freshly prepared Alamar blue (Himedia) was added to each well of the plate, and plates were further incubated at 37 °C for 30 minutes. After incubation, the fluorescence was measured at excitation/emission of 540–570/580–610 nm. The percentage inhibition and viability were calculated for the controls, and the Gompertz curve was utilized to estimate the minimum inhibitory concentration.

Further, the MBC of the compounds was determined by spreading 20 μL aliquots from wells, which showed no visible growth in MIC on Mueller–Hinton agar (MHA) plates, and the bacterial cells were enumerated after incubation at 37 °C for 24 hours. MBC is defined as the lowest concentration of the compound at which more than 99.9% of the cells were killed compared with the growth control. The minimum compound concentration at which no visible bacterial growth was observed was taken as the Minimum Bactericidal Concentration (MBC).^50^

### 3.8 Cell viability assay

The effect of compounds (IBC, IBC-2, and IBC-3) on the viability of cells was evaluated at different concentrations against macrophage cell lines through the 3-(4,5-dimethylthiazol2-yl)-2, 5-diphenyltetrazolium bromide (MTT) assay. The RAW 264.7 cells are monocyte/macrophage-like cells originating from an Abelson leukemia virus-transformed cell line derived from BALB/c mice. Due to their reliability and consistency, these cell lines are widely utilized for both *in vitro* and *in vivo* analyses.^52^ The MTT assay is a colorimetric assay used to measure the viability and cytotoxicity of cells. The cells (2 × 10^4^ cells per well) were grown at 37 °C with 5% CO_2_ for 48 hours, then exposed to compounds with different concentrations. The drug (camptothecin at 10 μM concentration), growth, DMSO, and media controls were used for comparable result determination. After 20–21 hours, 20 μL of MTT dye at 2.5 mg mL^−1^ concentration was added to each well and incubated for 4 hours. 100 μL DMSO solubilized the water-insoluble purple and white formazan produced.^53^ The formation of the formazan salt by mitochondrial dehydrogenases was measured at 570.0 nm after incubating for 20 min. The percentage cytotoxicity was calculated with respect to the untreated cells, and the CC_50_ value was calculated. CC_50_ was defined as the concentration of an inhibitor that reduces cellular activities by 50% compared to untreated control cultures. The CC_50_ values were calculated as the concentration where the absorbance was reduced by 50% using GraphPad Prism software.^54^ The selectivity index (SI) determines the safety profile of the compound. It is calculated as the ratio of compound concentration at which 50% of cell growth is inhibited compared to untreated cells (CC_50_) to the effective inhibiting concentration against bacterial strain (MIC). The high SI (>10 fold of MIC) of a drug is proven to have a favorable safety profile.^28^ Selectivity index (SI) = CC_50_ of the compound (μM)/MIC of the compound (μM).

### 3.9 Time-kill assay

Time-kill curve analyses were performed per CLSI (Clinical and Laboratory Standards Institute) guidelines. An overnight culture of *S. aureus* ATCC 25923 grown in Mueller Hinton broth (MHB) with turbidity equivalent to 0.5 McFarland standard was diluted 100 times, equivalent to 1.5 × 10^6^ CFU mL^−1^. The concentrations equal to MIC, twice the MIC, and four times the MIC of selected compounds were prepared in MHB, and an inoculum size of 1 × 10^6^ CFU mL^−1^ of standardized organism was added. Ten times the MIC of ciprofloxacin was used as the drug control, and untreated bacterial cells were used as the growth control. The cultures were then incubated in a shaker at 37 °C for 24 hours. Aliquots of 10 μL culture were removed at indicated time points of incubation, and serial dilutions were plated on MHA plates for the determination of viable cells (CFU mL^−1^).^55,56^

### 3.10 Combinational study

The synergistic effect of compounds (IBC, IBC-2, and IBC-3) with reference drugs ciprofloxacin and vancomycin against *S. aureus* was performed per the guidelines of the Clinical and Laboratory Standards Institute (CLSI). For the experiment, the compounds and selective antibiotics were dissolved in DMSO. In a 96-well flat bottom plate, the reference drug with 4× MIC was serially diluted horizontally. The test compounds with 4× MIC were serially diluted two-fold outside the plate in 1.5 mL Eppendorf's using Mueller Hinton Broth (MHB). These two serially diluted compounds were combined to create drug-compound combinations in the plate. The reference drug (horizontally) and tested compounds (vertically) alone were serially diluted for MIC determination. 100 μL of the standardized bacterial inoculum with a turbidity equivalent to a 0.5 McFarland standard were diluted 1 : 100 in Mueller Hinton Broth and inoculated in the plates. The plates were incubated for 24 hours at 37 °C. After incubation, 10 μL of freshly prepared Alamar blue (Himedia) was added to each well of the plate, and the plates were further incubated at 37 °C for 30 minutes. After incubation, the plates were visually observed. The Fractional Inhibitory Concentration Index (FICI) was calculated for each tested compound and reference drug to find a synergistic effect. The FICI was calculated using the formula FICI = FIC of compound + FIC of drug. The calculation followed is FICI (*C*_A_/(MIC_A_)) + (*C*_B_/(MIC_B_)), where *C*_A_ is the MIC of the compound in combination, *C*_B_ is the MIC of the drug in combination, and MIC_A_ and MIC_B_ are the MIC values of individual compounds and drugs, respectively. The range selection followed the interpretation criteria. The FICI range of 0.5 to 4 indicates indifference, ≤ or =0.5 indicates synergy and >4 indicates antagonism.^57^

### 3.11 To evaluate the effect of the IBC-3 on the morphological appearance of *S. aureus*

To observe the efficiency of the IBC-3 in targeting the cell integrity of *S. aureus*, scanning electron microscopy (SEM) was performed.^58^ Briefly, *S. aureus* was cultured in MHB, incubated at 37 °C for 3–4 hours, and diluted further to 0.5 OD at 600 nm. The culture was treated with different concentrations of the test compound, 1× MIC (10 μM) and 2× MIC (20 μM), while vancomycin at 4× MIC (2.8 μM) concentration was used as drug control. The untreated cells were used as growth control to compare the results. The treated cultures were then incubated at 37 °C for 6 hours. To prepare the sample, the culture was centrifuged and given phosphate-buffered saline of pH-7.4 (PBS) washings three times. The sample was then fixed overnight using step-by-step loading of 4% paraformaldehyde and 2.5% glutaraldehyde onto samples placed on gel bond film. Samples were dehydrated using a graded sequence of ethanol (30%, 50%, 70%, 80%, 90% and 100%).^59^ Completely dried samples were then mounted and coated with colloidal gold before the observation using a scanning electron microscope (EVO18 SEM Zeiss).

### 3.12 Cell integrity analysis

Propidium iodide (PI) indicates cell integrity, emitting a red fluorescence signal. PI's polar and hydrophilic nature impedes its entrance within active bacterial cells, but within perforated dead cells, it can easily bind to nucleic acid (DNA/RNA).^32,60^*S. aureus* bacterial cells were incubated overnight at 37 °C and diluted further 1 : 100 in Mueller Hinton Broth (MHB). The diluted culture was grown until its OD_600_ reached 0.5. The bacterial cells were treated with 1× MIC and 2× MIC of the IBC-3 compound for 30 minutes. Vancomycin was used as a drug control at 1× MIC, while untreated bacterial cells were used as growth control. The samples were centrifuged at 5000 rpm speed at 4 °C temperature for 10 minutes, and the pellet was washed two times using phosphate-buffered saline pH-7.4 (PBS). To stain the sample (dead cells), 3 μL PI of 20.0 mM concentration was added to the 1 mL solution (pellet resuspended in PBS) and incubated for 15 minutes. To remove the dye, the samples were centrifuged again and washed with PBS once. The final pellet was resuspended in 1 mL of PBS, and 30 μL of each undiluted sample was used to obtain fluorescence imaging. The cells were observed under fluorescence microscopy.^61^

### 3.13 *In silico* ADMET

The physicochemical and ADMET parameters for the active compounds were analyzed using the SwissADME software developed by the Swiss Institute of Bioinformatics.^62^ The physicochemical parameters include Lipinski's rule of 5, drug-likeness, and synthetic accessibility for the molecule of interest.^63^ The ADMET (absorption, distribution, metabolism, and excretion) parameters were also analyzed using the same tool. The structure of compounds was obtained using ChemDraw software and reconfirmed by ChemBio 3D Draw software. SMILES of compounds were generated from the Chemdraw software and then pasted into the SwissADME software, which gives us the structure of compounds. All physicochemical and ADMET parameters were generated using online software. The Bioavailability Radar depicts the drug-likeness of the compounds. The pink region delineates the optimal range across six parameters: molecular weight (150–500 g mol^−1^), lipophilicity (*X* log *P*3 between −0.7 and 6.0), solubility (log *S* ≤ 6), polarity (TPSA between 20–130 Å^2^), saturation (carbon fraction in sp^3^ hybridization ≥0.25), and flexibility (no more than 9 rotational bonds). Any deviation from these ranges represents suboptimal physicochemical properties for oral bioavailability. Additionally, the compounds were evaluated for their gastrointestinal (GI) absorption, potential to inhibit CYP450 isozymes, and permeability across the blood-brain barrier.

## 4 Conclusion

In our study, we found that prenylated chalcone (IBC) and its flavonone (IBC-2) and aurone (IBC-3) derivatives show antibacterial properties against clinically significant pathogens, including both Gram-positive (*S. aureus* and *MRSA*) and Gram-negative (*E. Coli* and *P. aeruginosa*) strains. The evaluated compounds, particularly IBC-3, demonstrated robust antimicrobial activity against a broad spectrum of bacterial strains, showcasing significant bactericidal effects. Despite IBC and IBC-2 exhibiting strong efficacy against *S. aureus* ATCC 29213, IBC-3 stood out for its potent and broad-spectrum antimicrobial activities, addressing diverse infections and potentially minimizing resistance development risks. Cell viability assessments on the murine RAW macrophage cell line revealed minimal to moderate cytotoxicity for IBC-2 and IBC-3, with compounds IBC and IBC-2 showing a favorable selectivity index (>10) with respect to *S. aureus* ATCC 29213.

Furthermore, combination studies with known antibiotics revealed a noteworthy synergistic effect between IBC-3 and vancomycin against *S. aureus*. Time- and concentration-dependent studies further supported the bactericidal nature of the compounds, as IBC, IBC-2, and IBC-3 exhibited concentration-dependent killing against *S. aureus* in a time-dependent manner. Given vancomycin's cell wall targeting mechanism, the complementary action of IBC-3 likely contributes to this synergistic impact, effectively inhibiting bacterial growth. The efficiency of this IBC-3 in altering the morphological appearance of *S. aureus*, observed in scanning electron microscopy (SEM) and propidium iodide (PI) staining studies, further confirms the prediction that it disintegrates the cytoplasmic membrane or cell wall and likely targets the cell wall for bacterial inhibition. These findings underscore the potential therapeutic value of this structurally diverse prenylated natural product, its derivatives, and the physicochemical properties requisite for their antibacterial efficacy, inhibiting antibacterial targets, providing insights into drug design, and developing novel antibiotic agents to combat resistant bacterial infections.

## Data availability

The data used in this study is in the ESI.†

## Conflicts of interest

There is no conflict of Interest.

## Supplementary Material

RA-014-D4RA05370B-s001
