# Supplementary material for: Antibacterial activity of structurally diverse natural prenylated isobavachalcone derivatives
Source: RSC Adv. 2024 Oct 17;14(45):32771–85. doi: 10.1039/d4ra05370b (PMC11484510; doi:10.1039/d4ra05370b)
Supplement: RA-014-D4RA05370B-s001 [file RA-014-D4RA05370B-s001.pdf]

Supplementary Information (SI) for RSC Advances.
This journal is © The Royal Society of Chemistry 2024

SUPPORTING INFORMATION

Antibacterial Activity of Structurally Diverse Natural Prenylated Isobavachalcone Derivatives

Puneet Kumar,[a,c] Sapna Saini,[b,c] Anjali Gangwar,[b,c] Rashmi Sharma,[b,c*] Jasha Momo H. Anal[a,c*]

[a]Natural Products and Medicinal Chemistry Division, CSIR– Indian Institute of Integrative Medicine, Jammu-180001, India.

[b]Infectious Division, CSIR–Indian Institute of Integrative Medicine, Jammu-180001, India.

[c]Academy of Scientific and Innovative Research (AcSIR), Ghaziabad - 201002, India

List of content

S. No.	List of Figures	Page No.
1.	IBC and synthesized compounds	3
2.	HPLC purity of IBC-1	4
3.	^{1}H NMR of IBC-1	5
4.	^{13}C NMR of IBC-1	5
5.	HRMS of IBC-1	6
6.	HPLC purity of IBC-2	7
7.	^{1}H NMR of IBC-2	8
8.	^{13}C NMR of IBC-2	8
9.	DEPT135 NMR of IBC-2	9
10.	HRMS of IBC-2	9
11.	HPLC purity of IBC-3	10
12.	^{1}H NMR of IBC-3	11
13.	^{13}C NMR of IBC-3	11
14.	DEPT135 NMR of IBC-3	12
15.	HRMS of IBC-3	12
16.	^{1}H NMR of IBC-4	13
17.	^{13}C NMR of IBC-4	13
18.	HRMS of IBC-4	14
19.	^{1}H NMR of IBC-5	15
20.	^{13}C NMR of IBC-5	15
21.	HRMS of IBC-5	16

22.	^{1}H NMR of IBC-6	17
23.	^{13}C NMR of IBC-6	17
24.	HRMS of IBC-6	18
25.	^{1}H NMR of IBC-7	19
26.	^{13}C NMR of IBC-7	19
27.	HRMS of IBC-7	20
28.	^{1}H NMR of IBC-8	21
29.	^{13}C NMR of IBC-8	21
30.	HRMS of IBC-8	22
31.	^{1}H NMR of IBC-9	23
32.	^{13}C NMR of IBC-9	23
33.	HRMS of IBC-9	24
34.	^{1}H NMR of IBC-10	25
35.	^{13}C NMR of IBC-10	25
36.	HRMS of IBC-10	26
37.	MBC of potent inhibitors against *S. aureus*, MRSA, *E. coli* and *P. aeruginosa*.	27

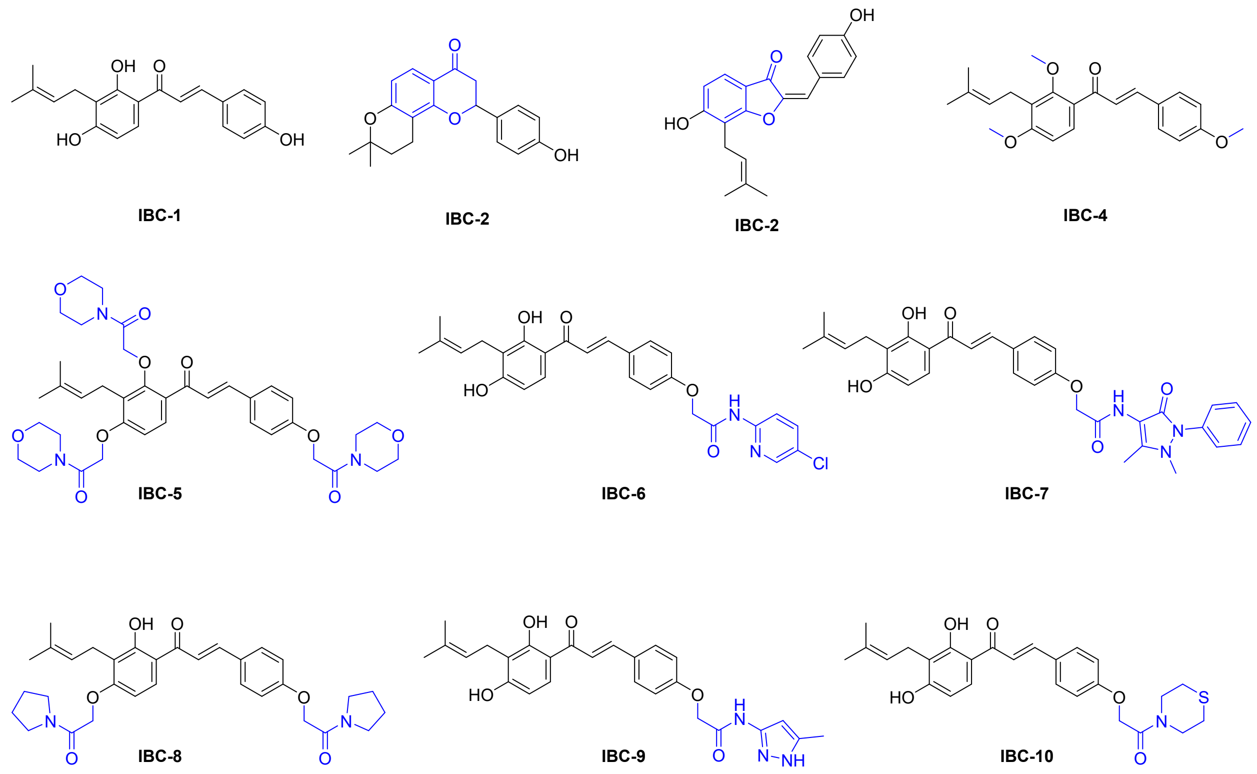

Figure 1. IBC and synthesized compounds

HPLC purity of IBC-1

SHIMADZU LabSolutions

Analysis Report

<Sample Information>

Sample Name	: ISB		
Sample ID	: ISB		
Data Filename	: 20-11-2023_02.lcd		
Method Filename	: 02-05-2023_Gradiant-04.lcm		
Batch Filename	: 20.11.2023-01.lcb		
Vial #	: 1-29	Sample Type	: Unknown
Injection Volume	: 5 uL		
Date Acquired	: 20/11/2023 12:47:22	Acquired by	: System Administrator
Date Processed	: 20/11/2023 14:13:14	Processed by	: System Administrator

<Chromatogram>

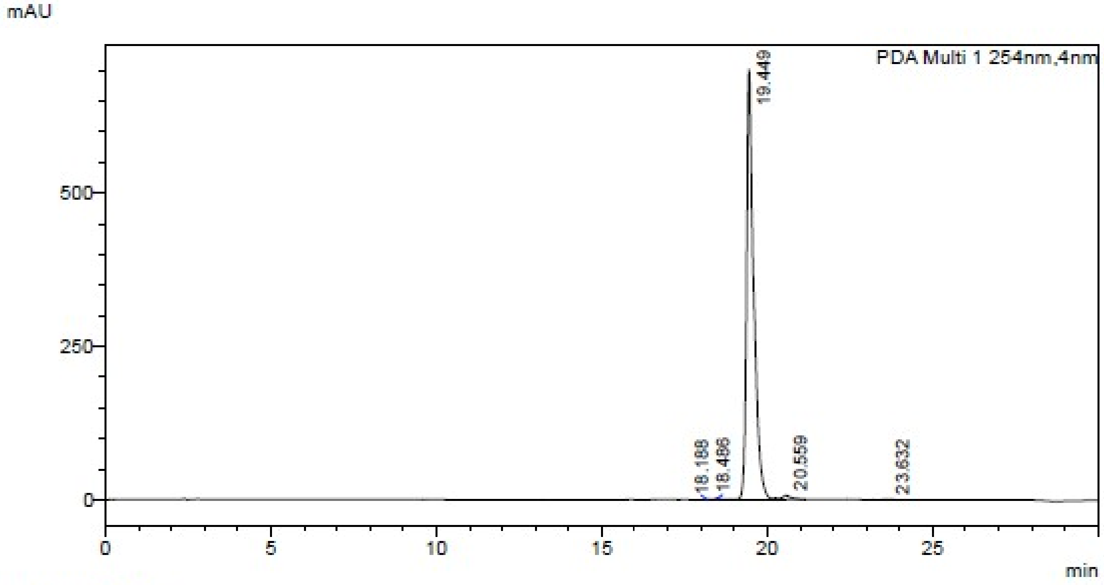

<Peak Table>

PDA Ch1 254nm

Peak#	Ret. Time	Area	Area%
1	18.188	7411	0.069
2	18.486	35302	0.328
3	19.449	10561885	98.187
4	20.559	141289	1.313
5	23.632	11041	0.103
Total		10756928	100.000

^{1}H NMR of IBC-1

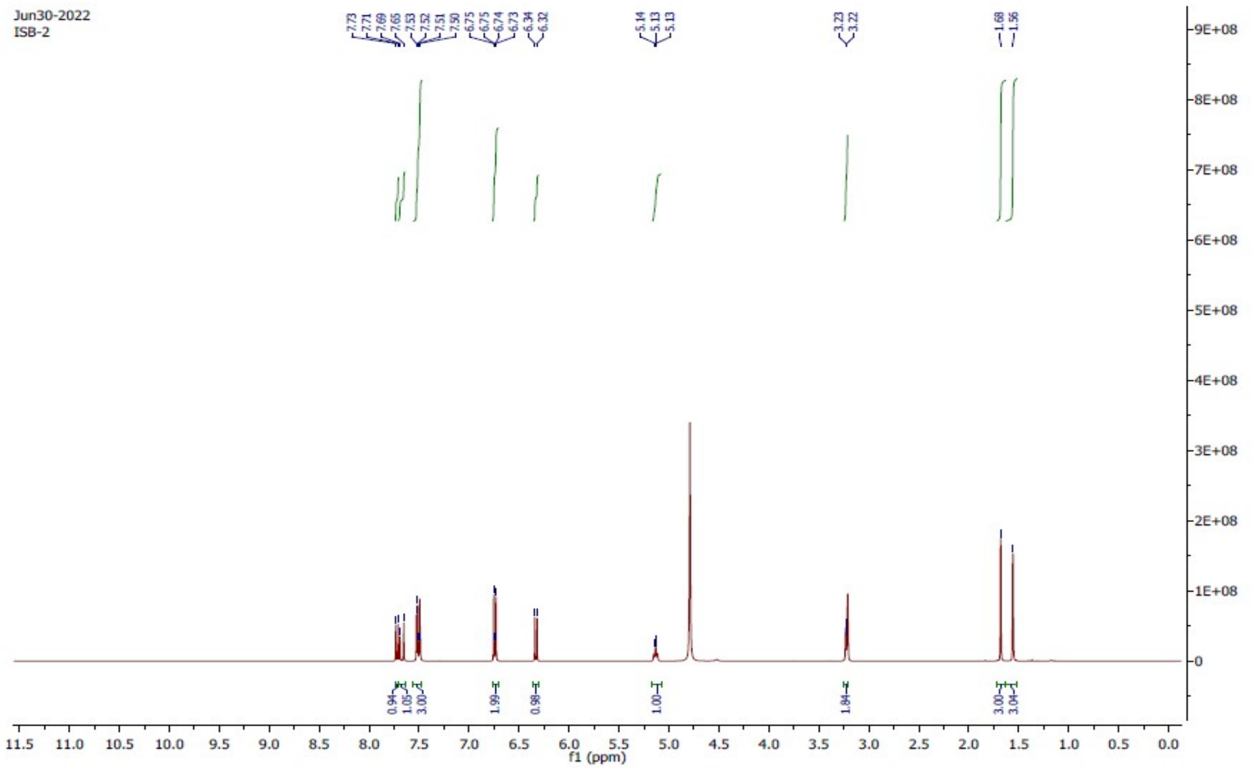

^{13}C NMR of IBC-1

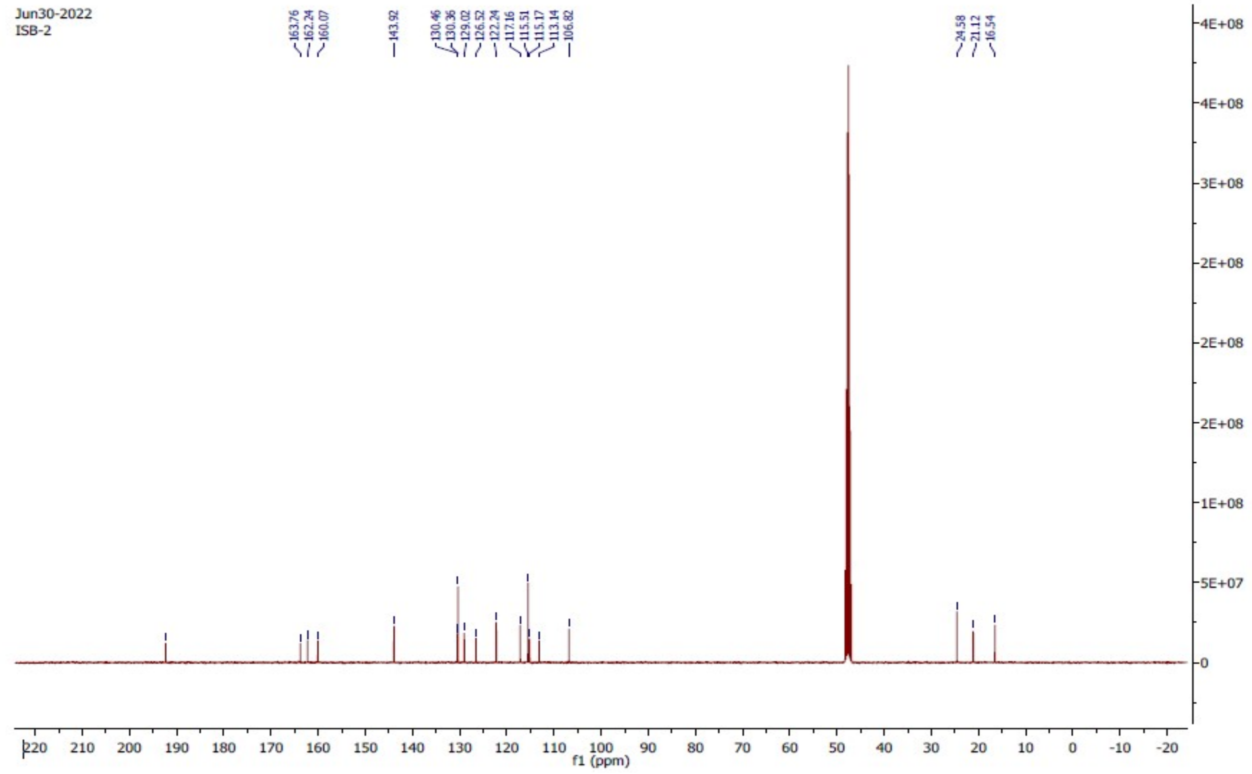

HRMS of IBC-1

Elemental Composition Report

Single Mass Analysis
Tolerance = 50.0 PPM / DBE: min = -1.5, max = 50.0
Element prediction: Off
Number of isotope peaks used for i-FIT = 3

Monoisotopic Mass, Even Electron Ions
8 formula(e) evaluated with 1 results within limits (up to 3 closest results for each mass)
Elements Used:
C: 0-20 H: 0-100 O: 0-4

ISB-2
QMI DIVISION, CSIR-IIIM JAMMU
Xevo G2-XS QTOF YFC2015
03-Jun-2022
12:18:14
030622_04 9 (0.208) Cm (9)
1: TOF MS ES+
1.87e+006

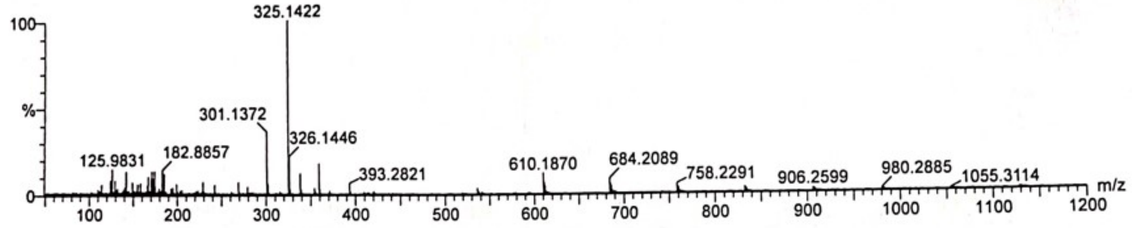

Minimum:				-1.5				
Maximum:		2.0	50.0	50.0				
Mass	Calc. Mass	mDa	PPM	DBE	i-FIT	Norm	Conf(%)	Formula
325.1422	325.1440	-1.8	-5.5	10.5	26.5	n/a	n/a	C20 H21 O4

HPLC purity of IBC-2

Natural Product Chemistry

USER:J.S.MOMO
Data file:C:\Documents and Settings\user\Desktop\PK-NEW\PK-ISB-2A.rslt\PK-ISB-2.dat
Method Name:C:\Documents and Settings\user\Desktop\PK-NEW\PK-ISB-2A.rslt\PK-N-2.met
5/3/2024 3:19:56 PM (GMT +05:30)
Sample ID:PK-ISB-2
Injection Vol.: 5

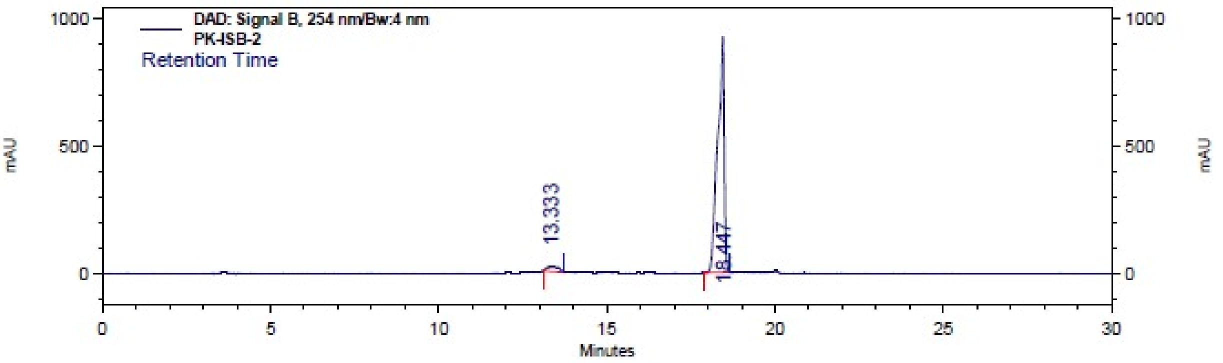

DAD:
Signal B,
254
nm/Bw:4 nm
Results

Peak Number	Retention Time	Area	Area Percent	Height	Height Percent
1	13.33	914693	3.34	36868	1.86
2	18.45	26471787	96.66	1942811	98.14
Totals		27386480	100.00	1979679	100.00

^{1}H NMR of IBC-2

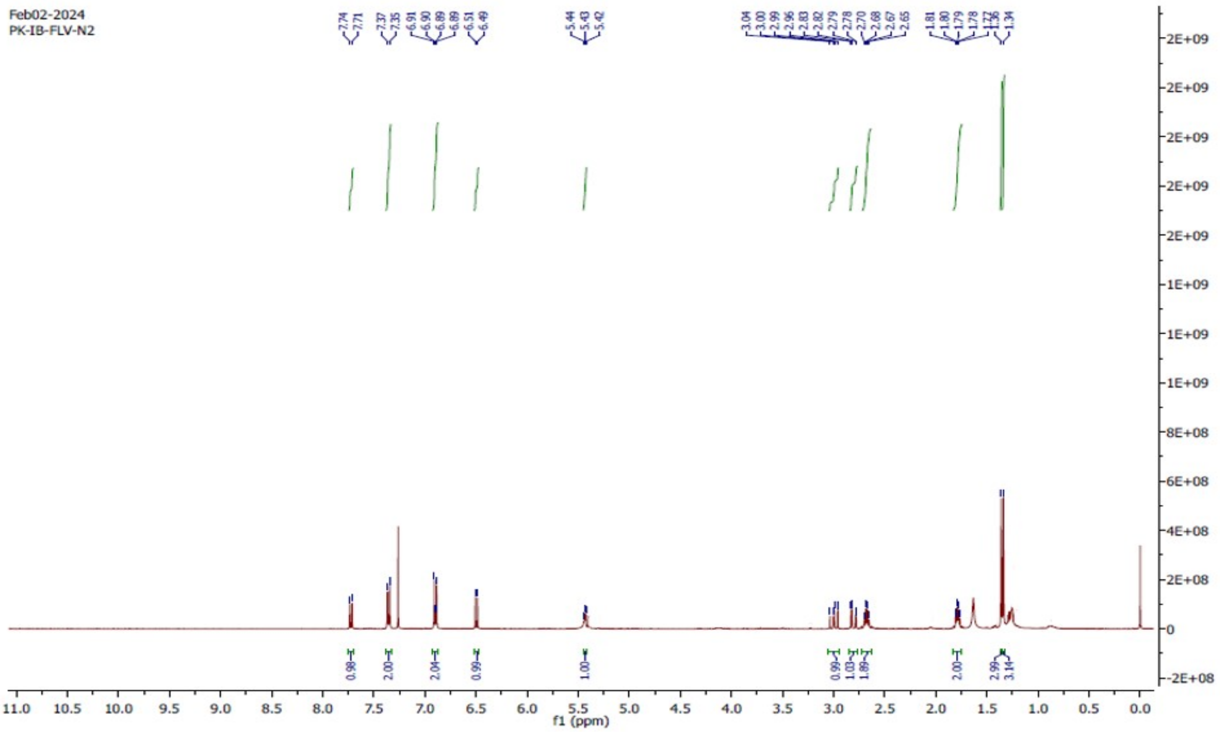

^{13}C NMR of IBC-2

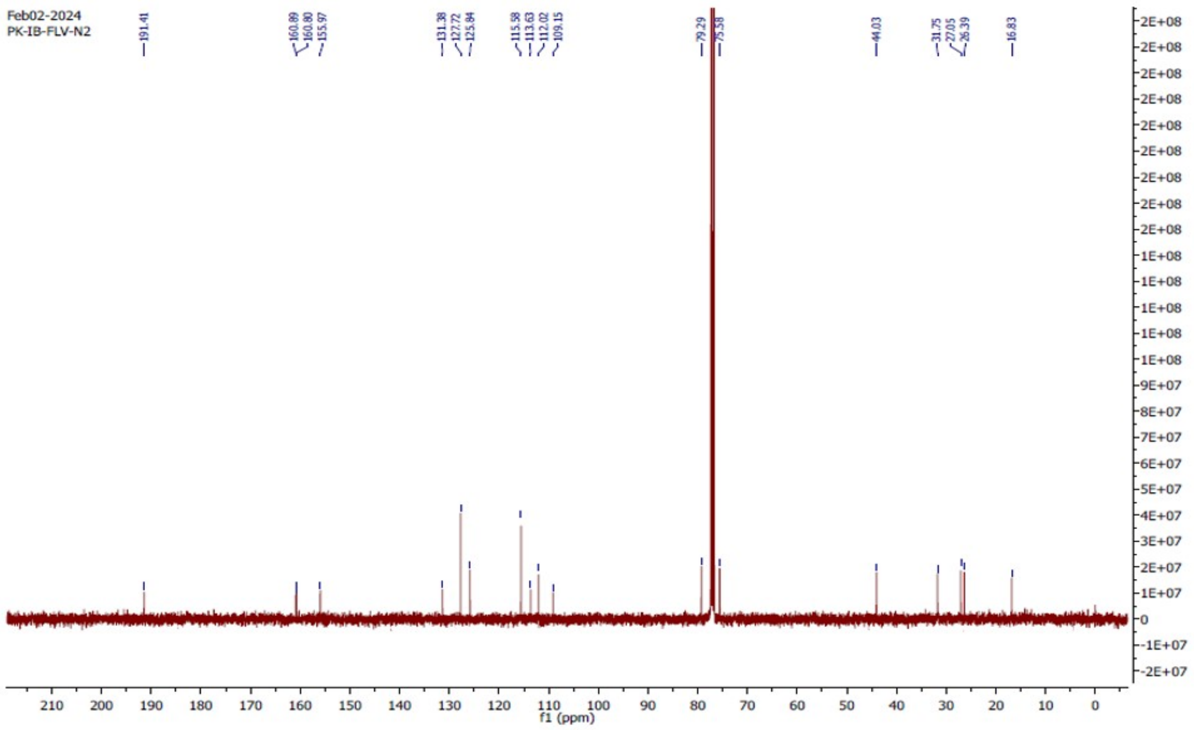

DEPT135 NMR of IBC-2

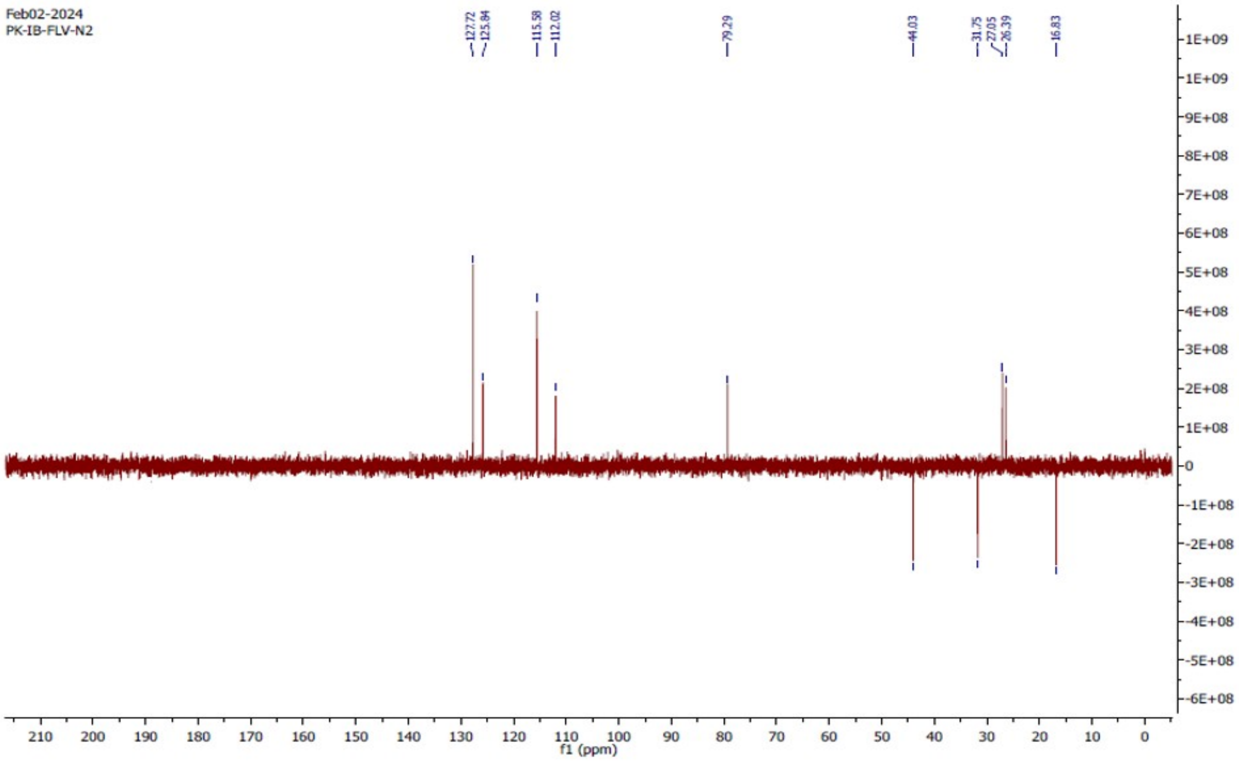

HRMS of IBC-2

Elemental Composition Report **Page 1**

Single Mass Analysis
Tolerance = 10.0 PPM / DBE: min = -1.5, max = 50.0
Element prediction: Off
Number of isotope peaks used for i-FIT = 3

Monoisotopic Mass, Even Electron Ions
8 formula(e) evaluated with 1 results within limits (up to 3 closest results for each mass)
Elements Used:
C: 0-20 H: 0-100 O: 0-4

PK-ISB-FLV-N2
QMI DIVISION, CSIR-IIIM JAMMU
Xevo G2-XS QTOF YFC2015
22-Feb-2024
12:49:25
220224_11 7 (0.155)
1: TOF MS ES+
1.63e+008

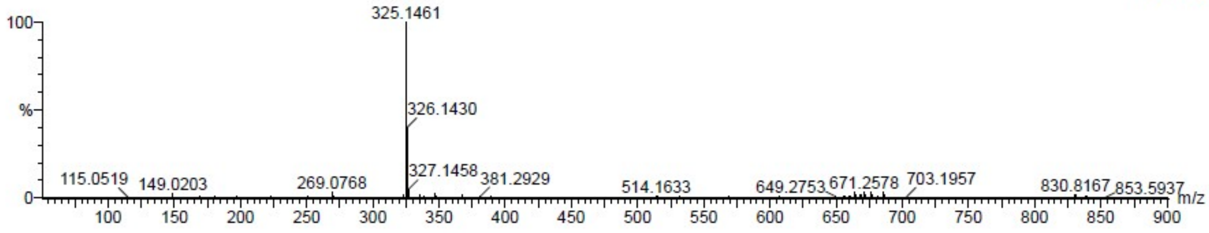

Minimum:				-1.5				
Maximum:		2.0	10.0	50.0				
Mass	Calc. Mass	mDa	PPM	DBE	i-FIT	Norm	Conf(%)	Formula
325.1461	325.1440	2.1	6.5	10.5	1417.6	n/a	n/a	C20 H21 O4

HPLC purity of IBC-3

Natural Product Chemistry

USER:J.S.MOMO
Data file:C:\Documents and
Settings\user\Desktop\PK-NEW\PK-ISB-AUR.rslt\PK-ISB-AUR.dat
Method Name:C:\Documents and
Settings\user\Desktop\PK-NEW\PK-ISB-AUR.rslt\PK-N.met
4/29/2024 11:20:56 AM (GMT +05:30)
Sample ID:PK-ISB-AUR
Injection Vol.: 5

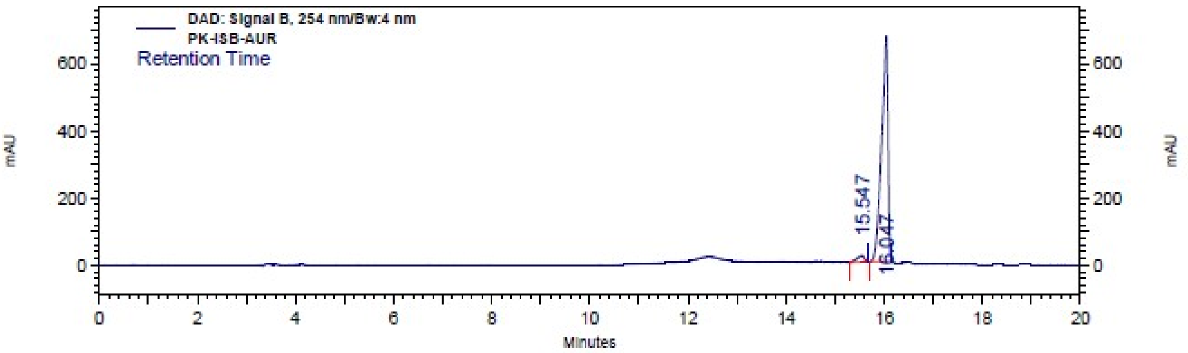

DAD:
Signal B,
254
nm/Bw:4 nm
Results

Peak Number	Retention Time	Area	Area Percent	Height	Height Percent
1	15.55	428589	3.24	39516	2.73
2	16.05	12809207	96.76	1410232	97.27
Totals		13237796	100.00	1449748	100.00

^{1}H NMR of IBC-3

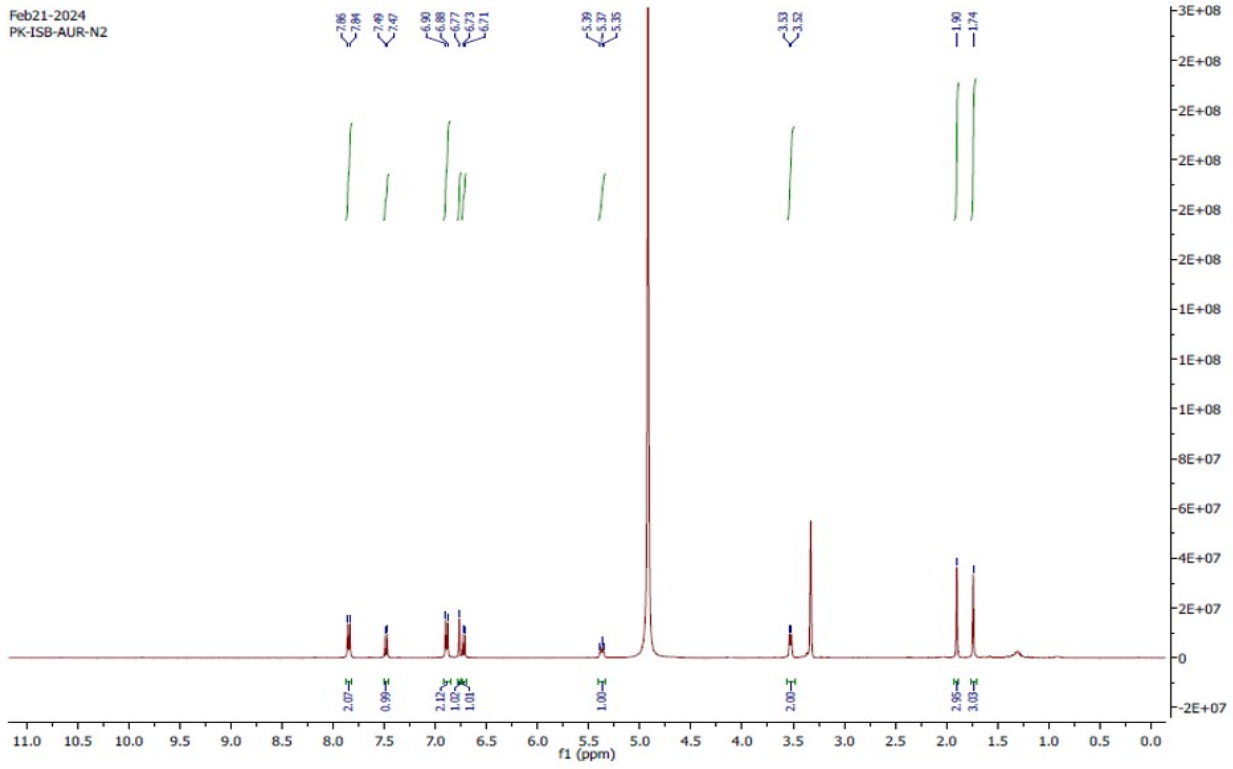

^{13}C NMR of IBC-3

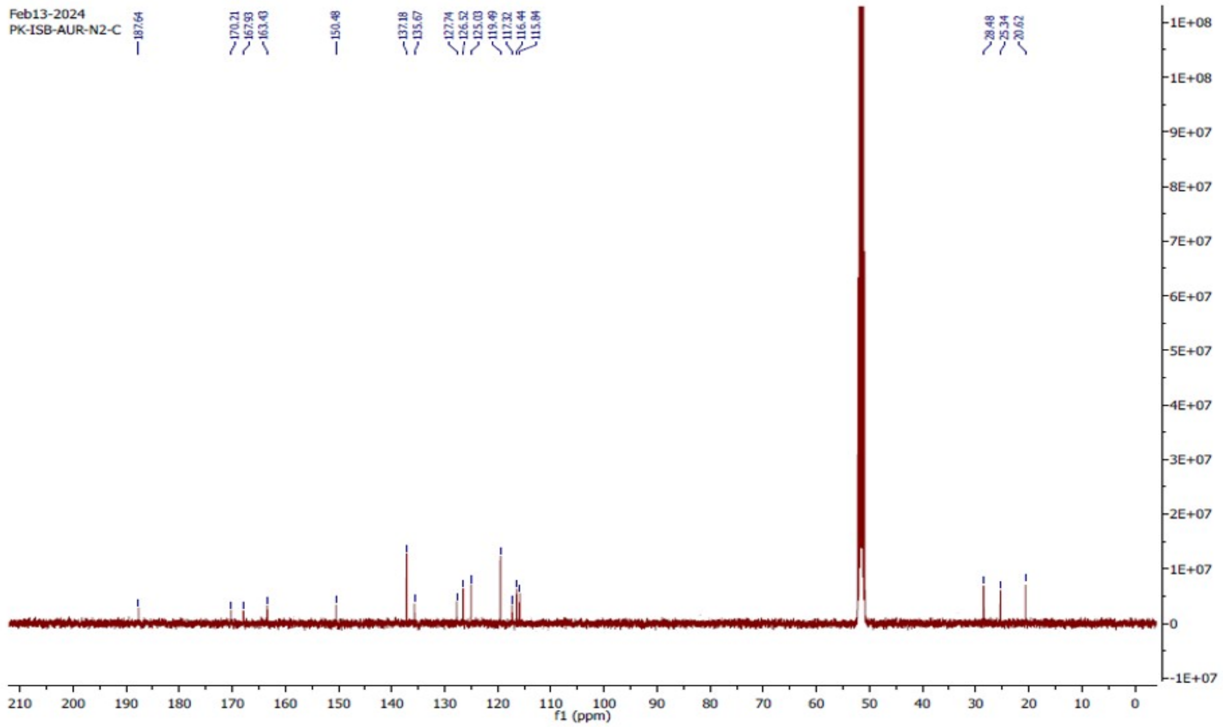

DEPT135 NMR of IBC-3

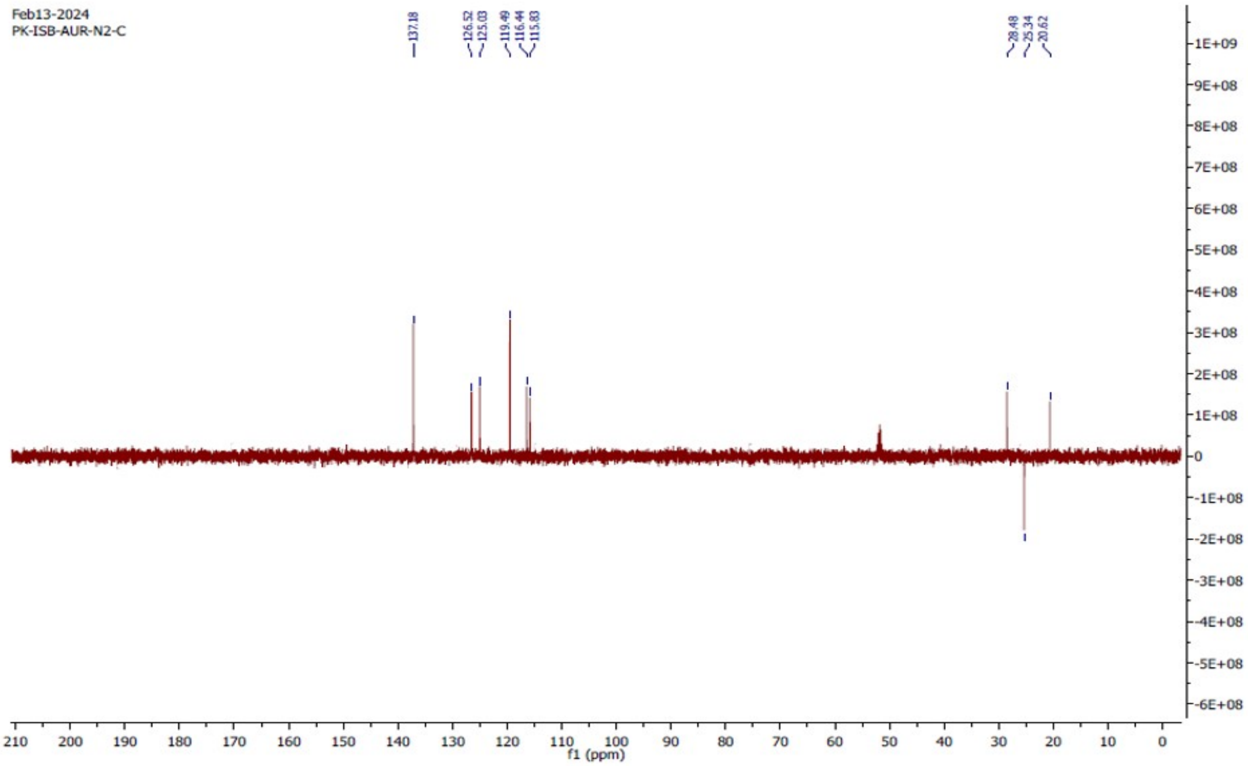

HRMS of IBC-3

Elemental Composition Report **Page 1**

Single Mass Analysis
Tolerance = 10.0 PPM / DBE: min = -1.5, max = 50.0
Element prediction: Off
Number of isotope peaks used for i-FIT = 3

Monoisotopic Mass, Even Electron Ions
8 formula(e) evaluated with 1 results within limits (up to 3 closest results for each mass)
Elements Used:
C: 0-20 H: 0-100 O: 0-4

PK-ISB-AUR-N2 QMI DIVISION, CSIR-IIIM JAMMU 22-Feb-2024
Xevo G2-XS QTOF YFC2015 12:52:07
220224_12 8 (0.172) 1: TOF MS ES+
2.88e+007

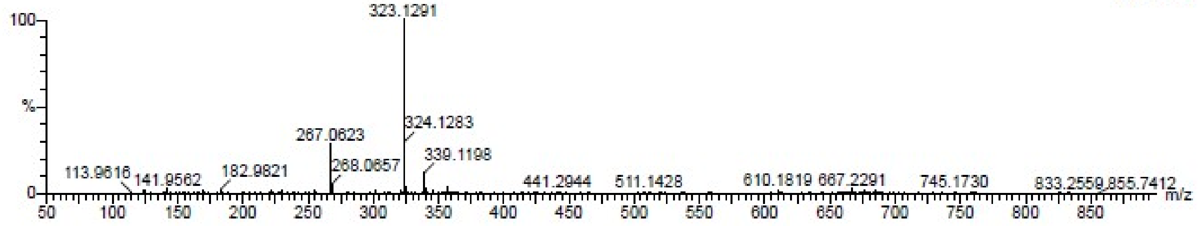

Minimum:				-1.5				
Maximum:		2.0	10.0	50.0				
Mass	Calc. Mass	mDa	PPM	DBE	i-FIT	Norm	Conf(%)	Formula
323.1291	323.1283	0.8	2.5	11.5	1347.6	n/a	n/a	C20 H19 O4

^{1}H NMR of IBC-4

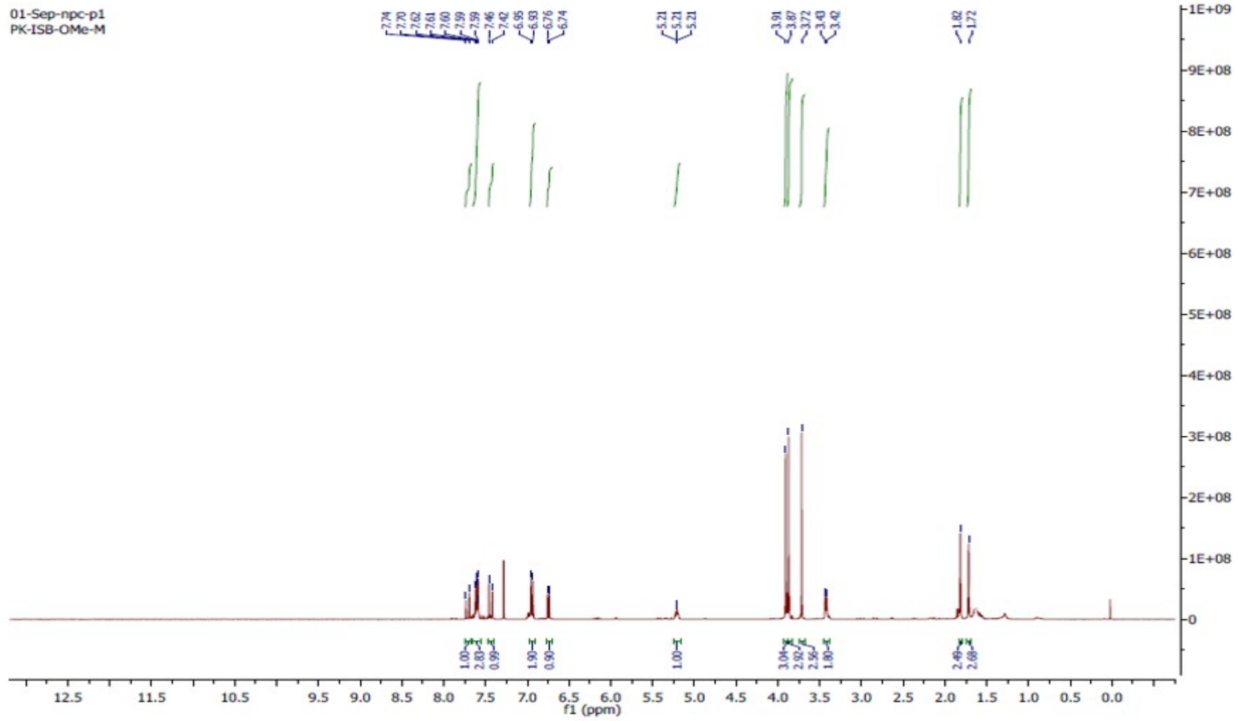

^{13}C NMR of IBC-4

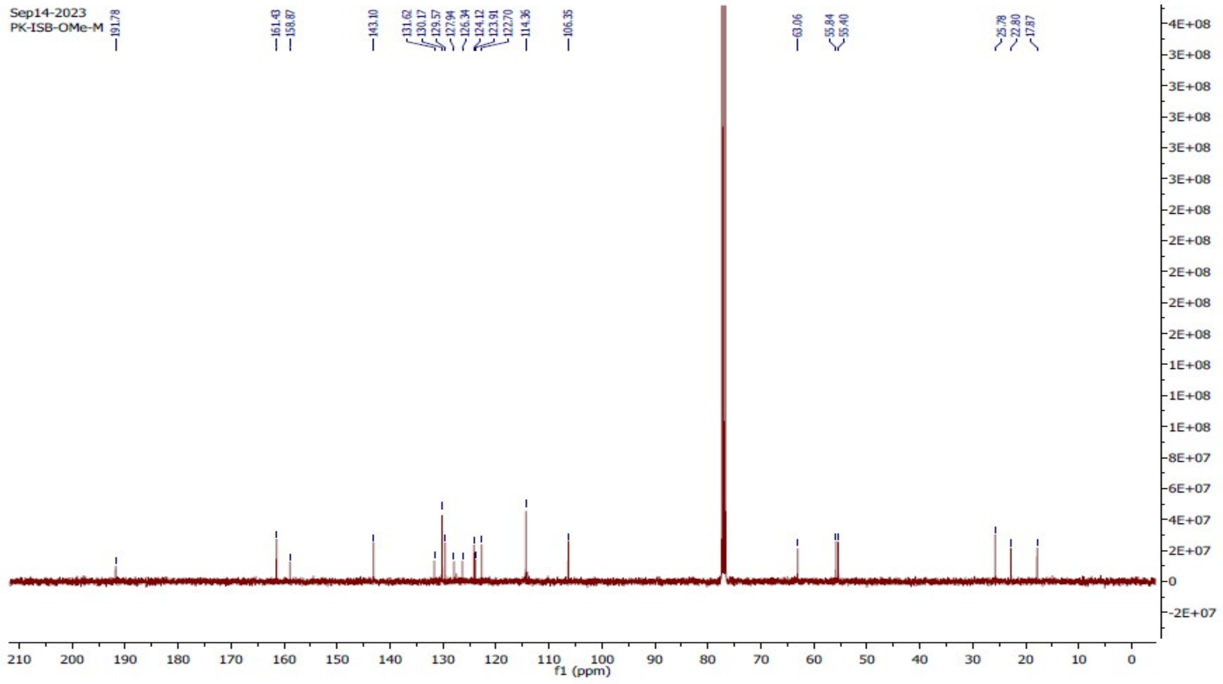

HRMS of IBC-4

Elemental Composition Report **Page 1**

Single Mass Analysis
Tolerance = 50.0 PPM / DBE: min = -1.5, max = 50.0
Element prediction: Off
Number of isotope peaks used for i-FIT = 3

Monoisotopic Mass, Even Electron Ions
8 formula(e) evaluated with 1 results within limits (up to 3 closest results for each mass)
Elements Used:
C: 0-23 H: 0-100 O: 0-4

PK-ISB-OMe-M
QMI DIVISION, CSIR-IIIM JAMMU
Xevo G2-XS QTOF YFC2015
06-Sep-2023
13:25:17
060923_09 4 (0.104)
1: TOF MS ES+
4.33e+006

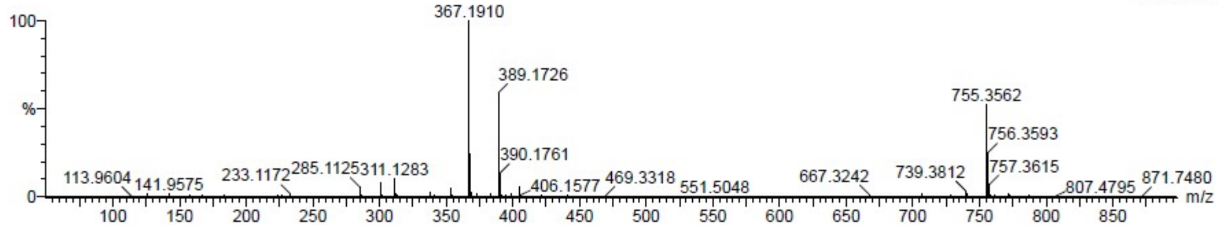

Minimum:				-1.5				
Maximum:		2.0	50.0	50.0				
Mass	Calc. Mass	mDa	PPM	DBE	i-FIT	Norm	Conf(%)	Formula
367.1910	367.1909	0.1	0.3	10.5	953.5	n/a	n/a	C23 H27 O4

^{1}H NMR of IBC-5

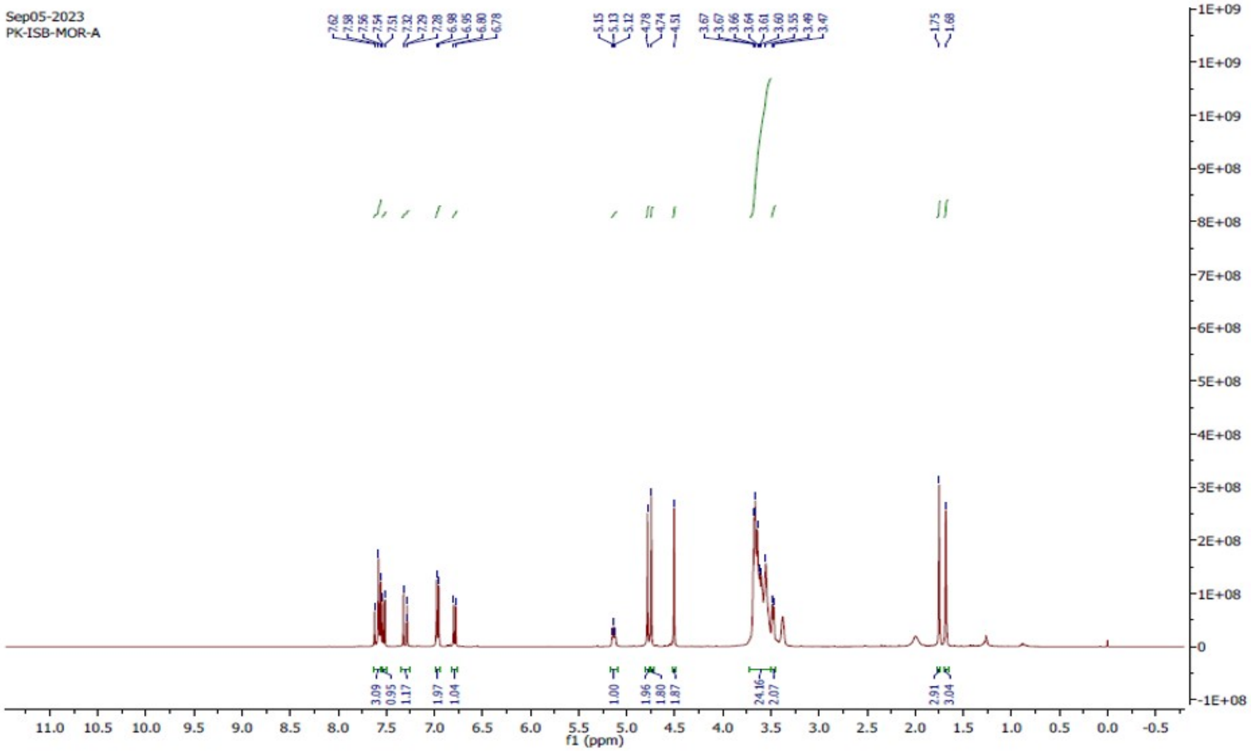

^{13}C NMR of IBC-5

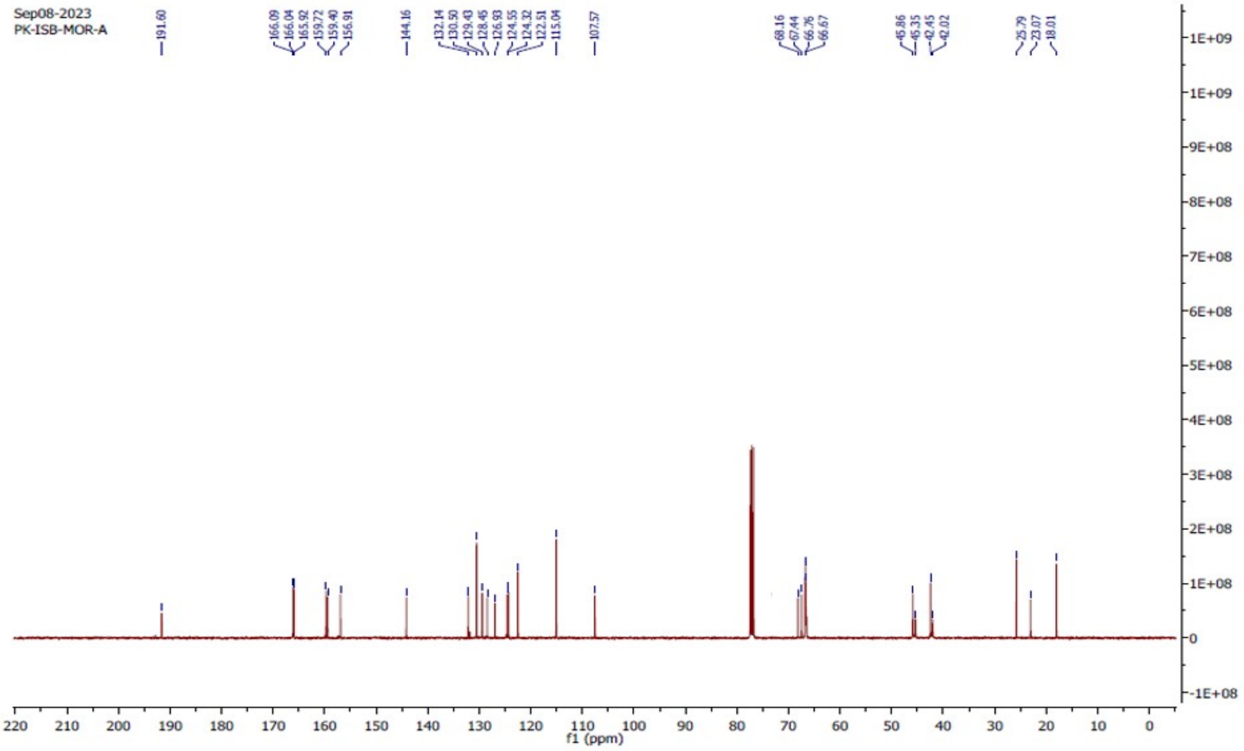

HRMS of IBC-5

Elemental Composition Report **Page 1**

Single Mass Analysis
Tolerance = 50.0 PPM / DBE: min = -1.5, max = 50.0
Element prediction: Off
Number of isotope peaks used for i-FIT = 3

Monoisotopic Mass, Even Electron Ions
50 formula(e) evaluated with 1 results within limits (up to 3 closest results for each mass)
Elements Used:
C: 0-38 H: 0-100 N: 0-3 O: 0-10

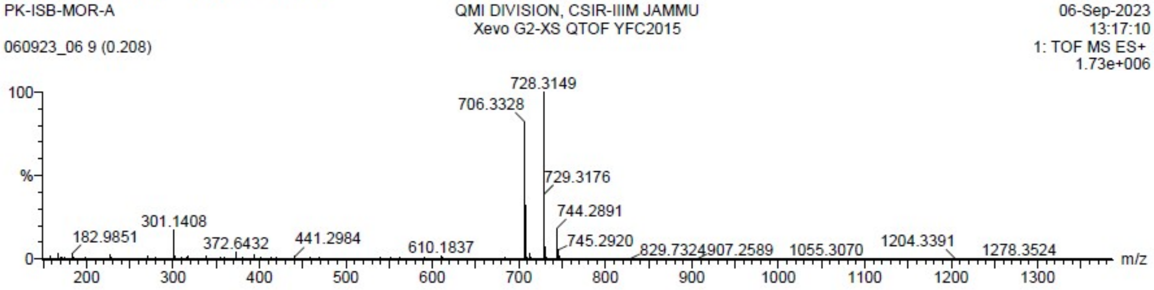

Minimum:				-1.5				
Maximum:		2.0	50.0	50.0				
Mass	Calc. Mass	mDa	PPM	DBE	i-FIT	Norm	Conf(%)	Formula
706.3328	706.3340	-1.2	-1.7	16.5	575.8	n/a	n/a	C38 H48 N3 O10

^{1}H NMR of IBC-6

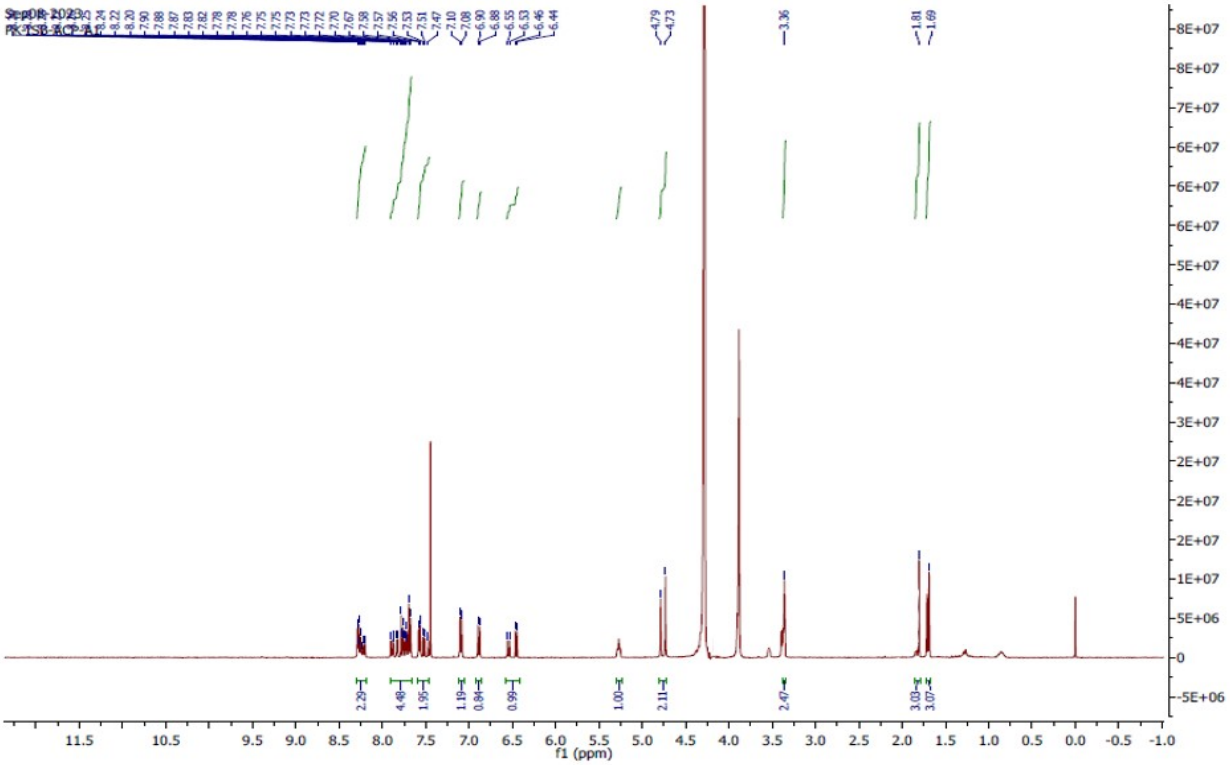

^{13}C NMR of IBC-6

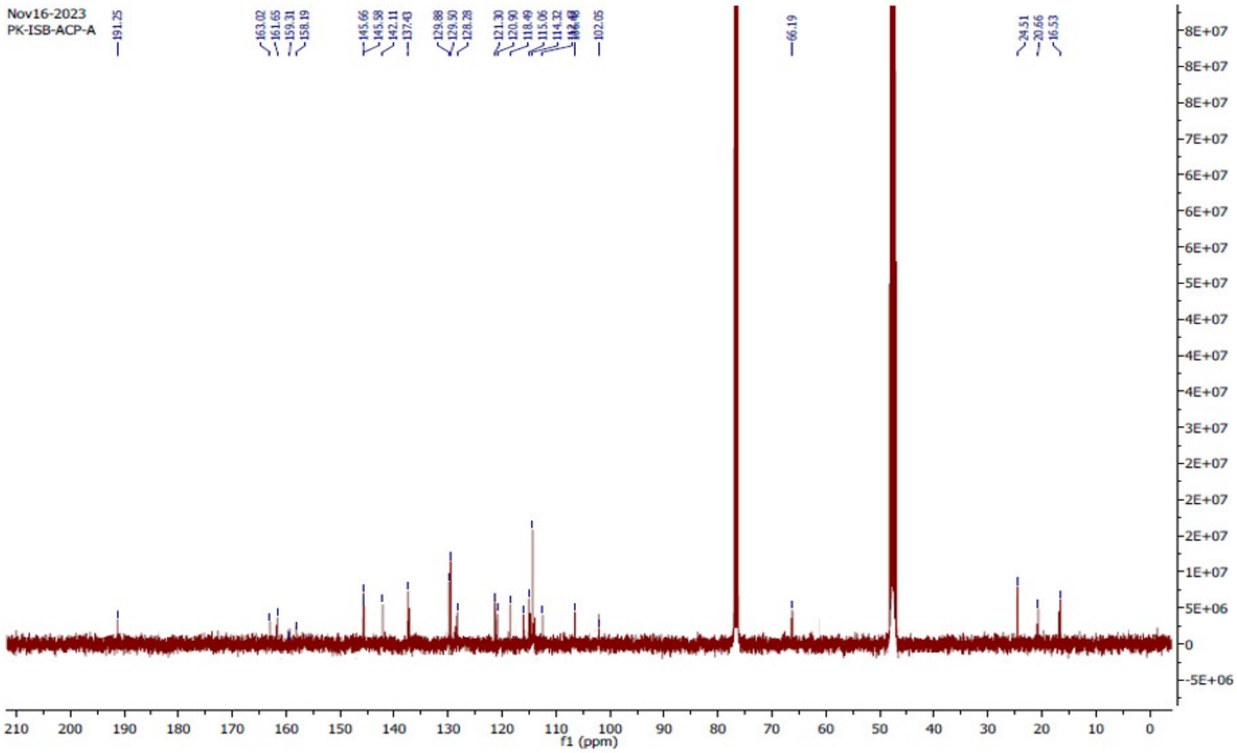

HRMS of IBC-6

Elemental Composition Report **Page 1**

Single Mass Analysis
Tolerance = 100.0 PPM / DBE: min = -1.5, max = 50.0
Element prediction: Off
Number of isotope peaks used for i-FIT = 3

Monoisotopic Mass, Even Electron Ions
40 formula(e) evaluated with 1 results within limits (up to 3 closest results for each mass)
Elements Used:
C: 0-27 H: 0-100 N: 0-2 O: 0-5 Cl: 0-1

PK-ISB-ACP QMI DIVISION, CSIR-IIIM JAMMU 11-Jan-2024
Xevo G2-XS QTOF YFC2015 15:17:08
110124_04 4 (0.104) 1: TOF MS ES+
1.66e+006

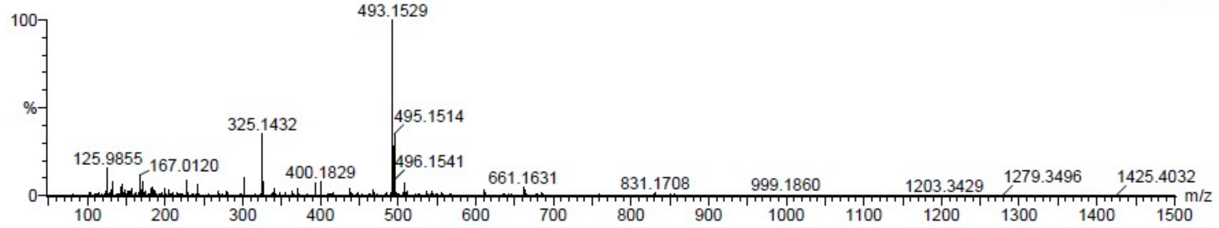

Minimum:				-1.5				
Maximum:		2.0	100.0	50.0				

Mass	Calc. Mass	mDa	PPM	DBE	i-FIT	Norm	Conf(%)	Formula
493.1529	493.1530	-0.1	-0.2	15.5	888.7	n/a	n/a	C27 H26 N2 O5 Cl

^{1}H NMR of IBC-7

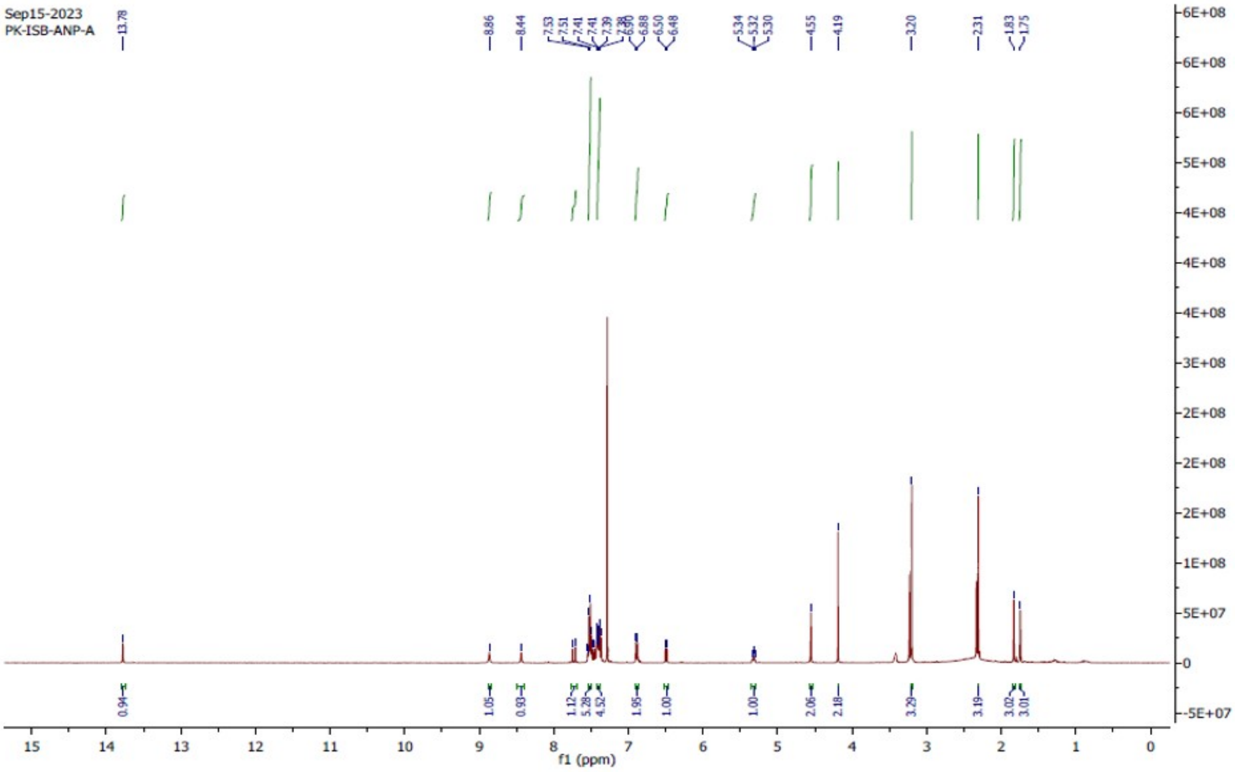

^{13}C NMR of IBC-7

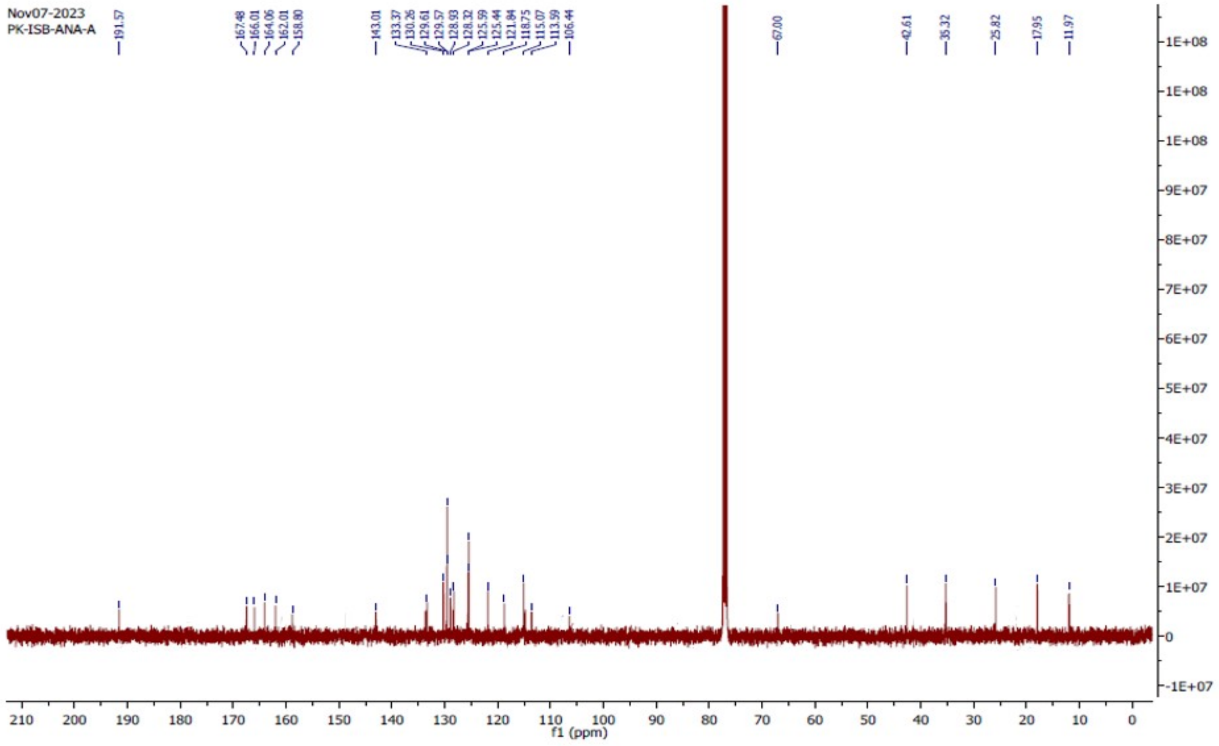

HRMS of IBC-7

Elemental Composition Report

Page 1

Single Mass Analysis
Tolerance = 100.0 PPM / DBE: min = -1.5, max = 50.0
Element prediction: Off
Number of isotope peaks used for i-FIT = 3

Monoisotopic Mass, Even Electron Ions
38 formula(e) evaluated with 1 results within limits (up to 3 closest results for each mass)
Elements Used:
C: 0-33 H: 0-100 N: 0-3 O: 0-6

PK-ISB-ANP | QMI DIVISION, CSIR-IIIM JAMMU | 11-Jan-2024
Xevo G2-XS QTOF YFC2015 | 15:24:50
110124_07 4 (0.104) | 1: TOF MS ES+ | 7.22e+006

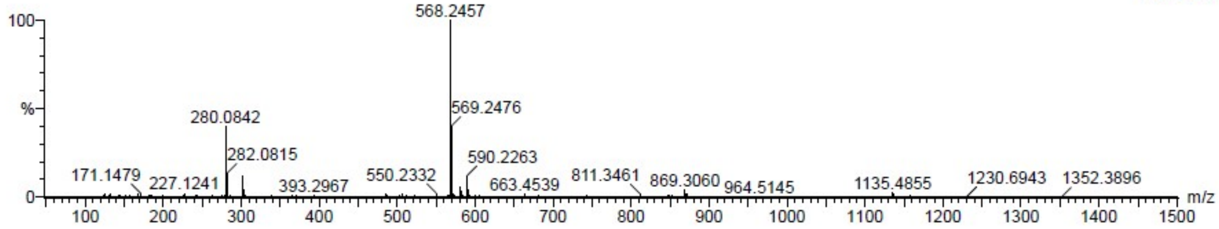

Minimum:				-1.5				
Maximum:		2.0	100.0	50.0				
Mass	Calc. Mass	mDa	PPM	DBE	i-FIT	Norm	Conf(%)	Formula
568.2457	568.2448	0.9	1.6	18.5	798.0	n/a	n/a	C33 H34 N3 O6

^{1}H NMR of IBC-8

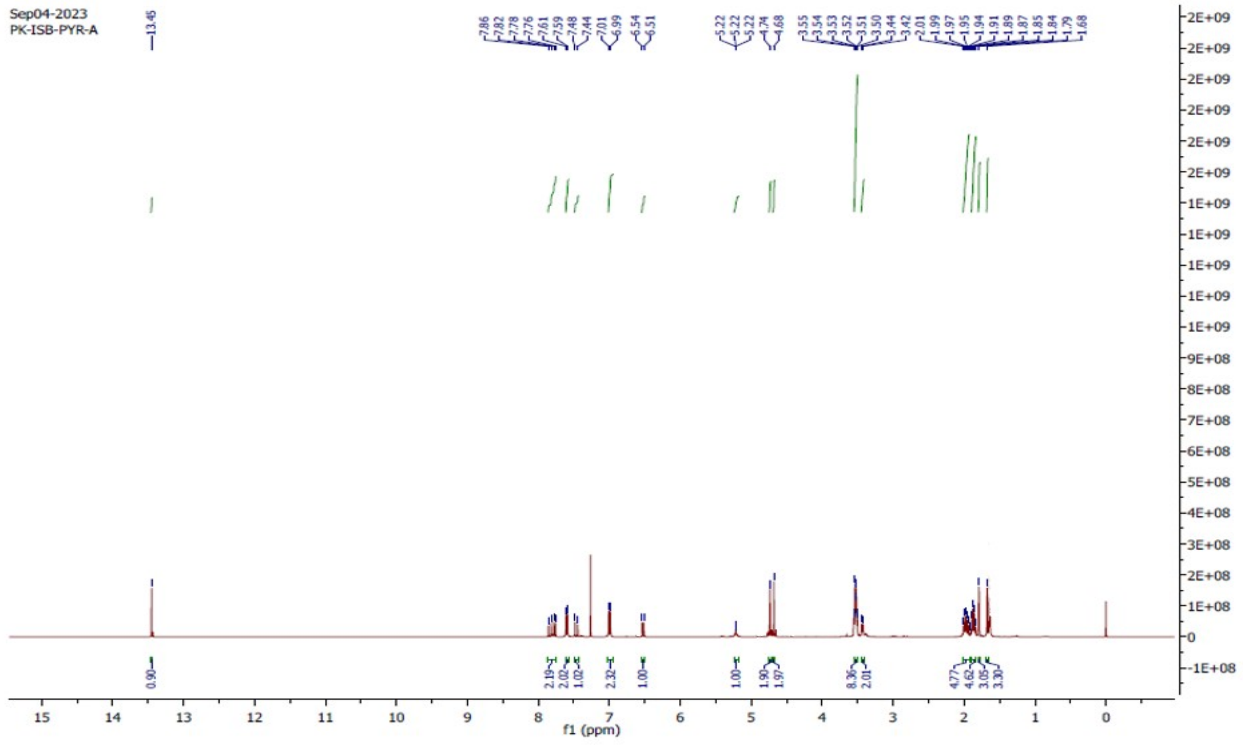

^{13}C NMR of IBC-7

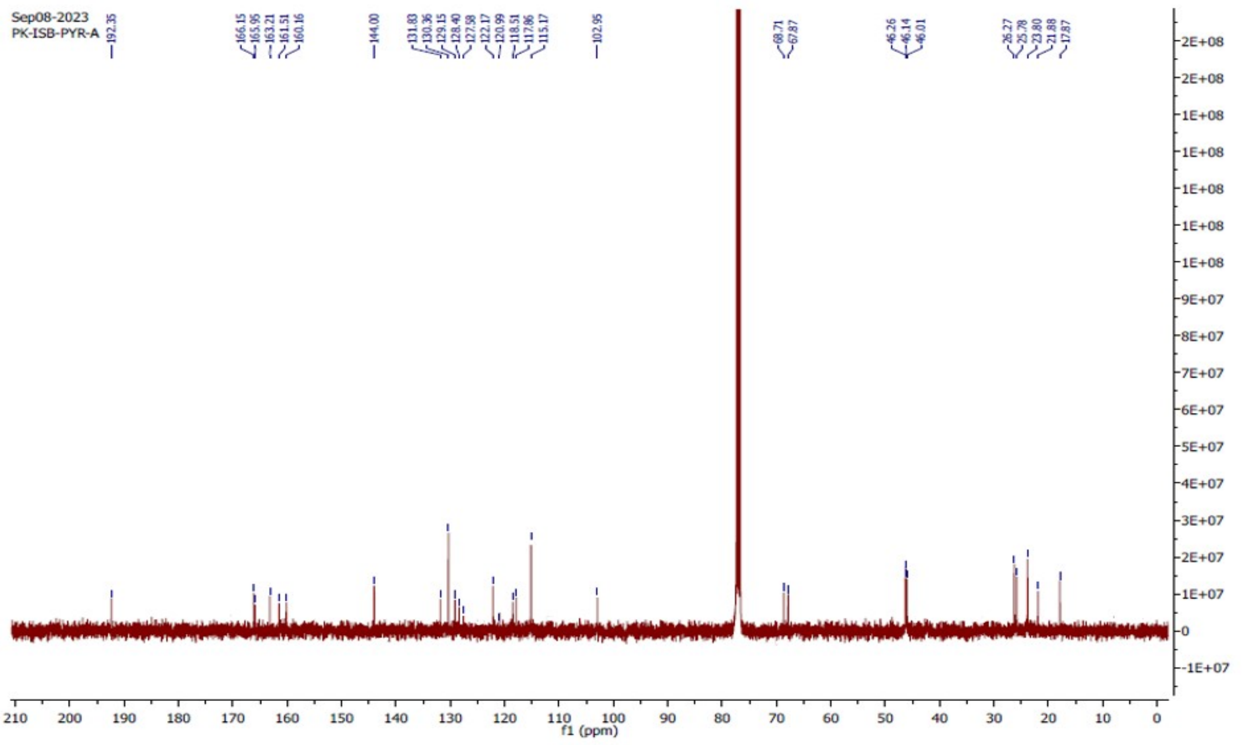

HRMS of IBC-8

Elemental Composition Report **Page 1**

Single Mass Analysis
Tolerance = 50.0 PPM / DBE: min = -1.5, max = 50.0
Element prediction: Off
Number of isotope peaks used for i-FIT = 3

Monoisotopic Mass, Even Electron Ions
25 formula(e) evaluated with 1 results within limits (up to 3 closest results for each mass)
Elements Used:
C: 0-32 H: 0-100 N: 0-2 O: 0-6

PK-ISB-PYR-A QMI DIVISION, CSIR-IIIM JAMMU 06-Sep-2023
Xevo G2-XS QTOF YFC2015 13:19:43
060923_07 8 (0.172) 1: TOF MS ES+
2.67e+006

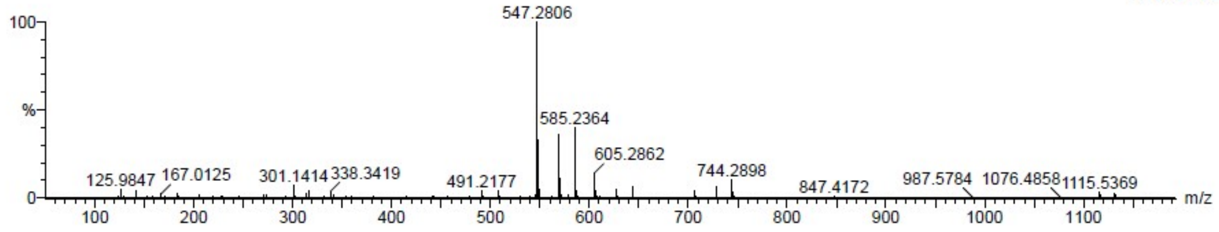

Minimum:				-1.5				
Maximum:		2.0	50.0	50.0				
Mass	Calc. Mass	mDa	PPM	DBE	i-FIT	Norm	Conf(%)	Formula
547.2806	547.2808	-0.2	-0.4	14.5	752.5	n/a	n/a	C32 H39 N2 O6

^{1}H NMR of IBC-9

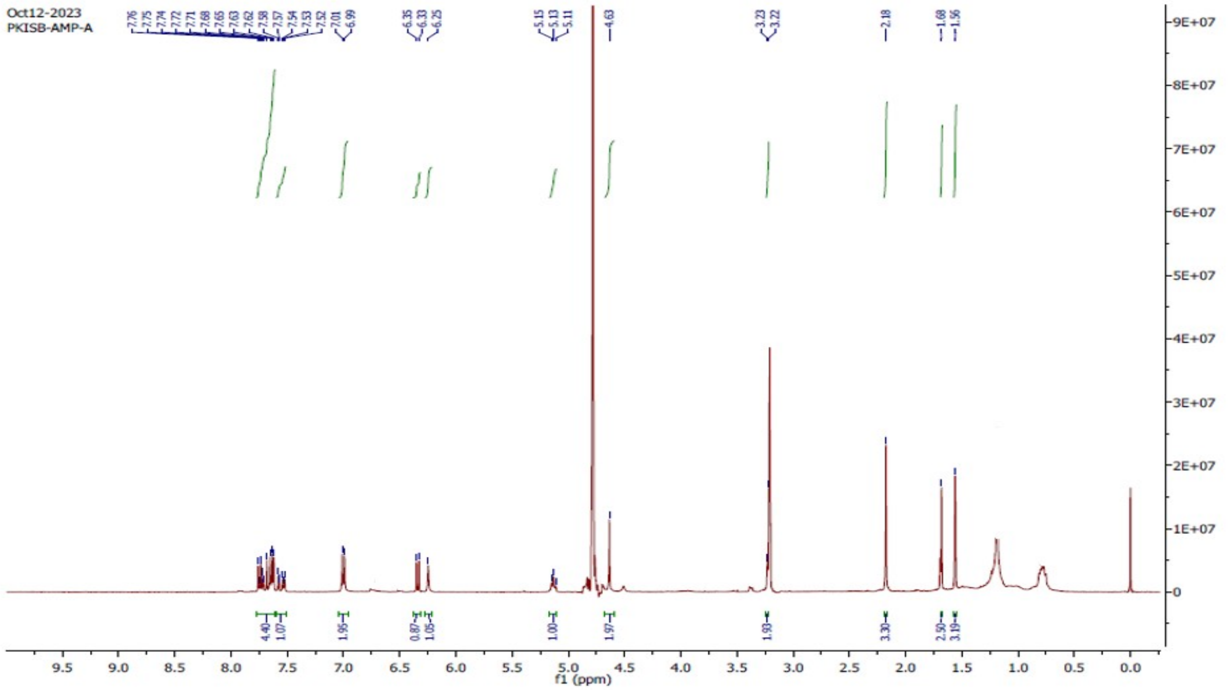

^{13}C NMR of IBC-9

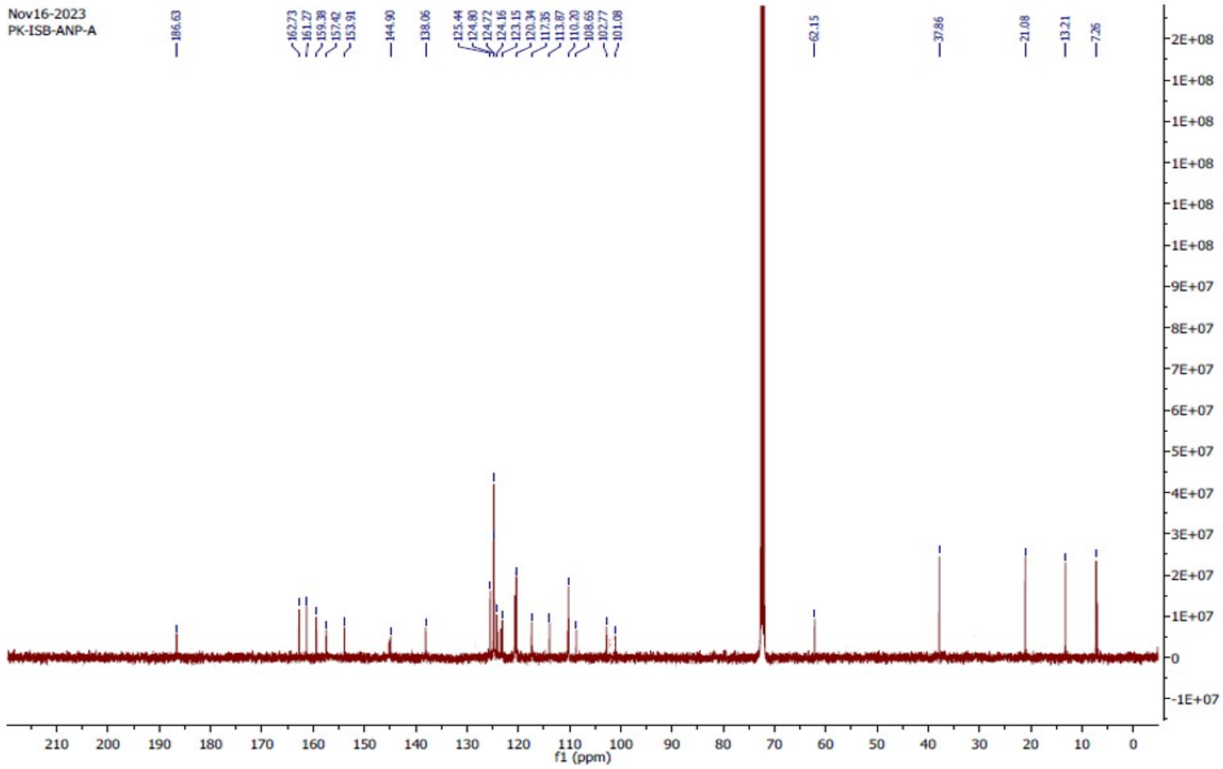

HRMS of IBC-9

Elemental Composition Report **Page 1**

Single Mass Analysis
Tolerance = 100.0 PPM / DBE: min = -1.5, max = 50.0
Element prediction: Off
Number of isotope peaks used for i-FIT = 3

Monoisotopic Mass, Even Electron Ions
28 formula(e) evaluated with 1 results within limits (up to 3 closest results for each mass)
Elements Used:
C: 0-26 H: 0-100 N: 0-3 O: 0-5

PK-ISB-AMP | QMI DIVISION, CSIR-IIIM JAMMU | 11-Jan-2024
Xevo G2-XS QTOF YFC2015 | 15:19:42
110124_05 8 (0.172) | 1: TOF MS ES+ | 3.00e+007

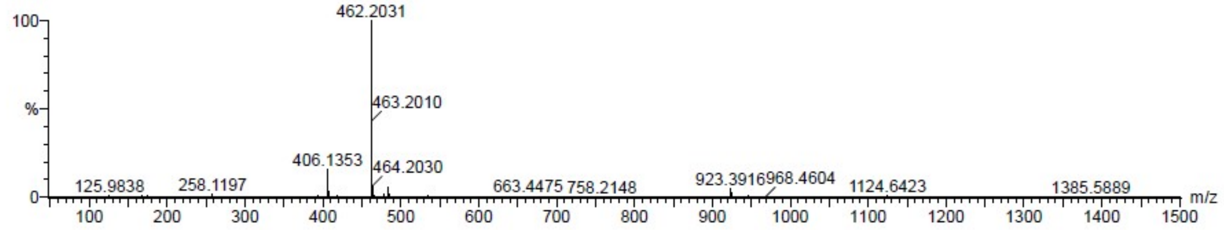

Minimum:				-1.5				
Maximum:		2.0	100.0	50.0				
Mass	Calc. Mass	mDa	PPM	DBE	i-FIT	Norm	Conf(%)	Formula
462.2031	462.2029	0.2	0.4	14.5	1022.3	n/a	n/a	C26 H28 N3 O5

^{1}H NMR of IBC-10

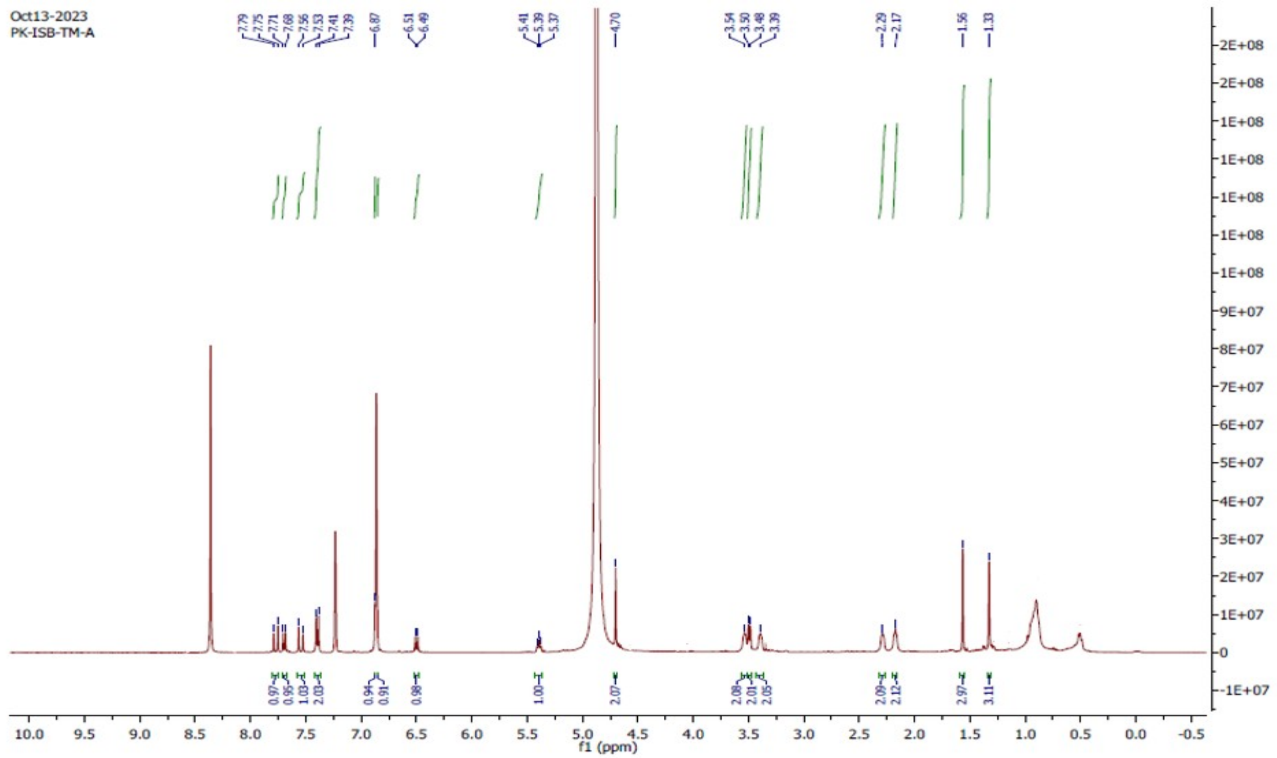

^{13}C NMR of IBC-10

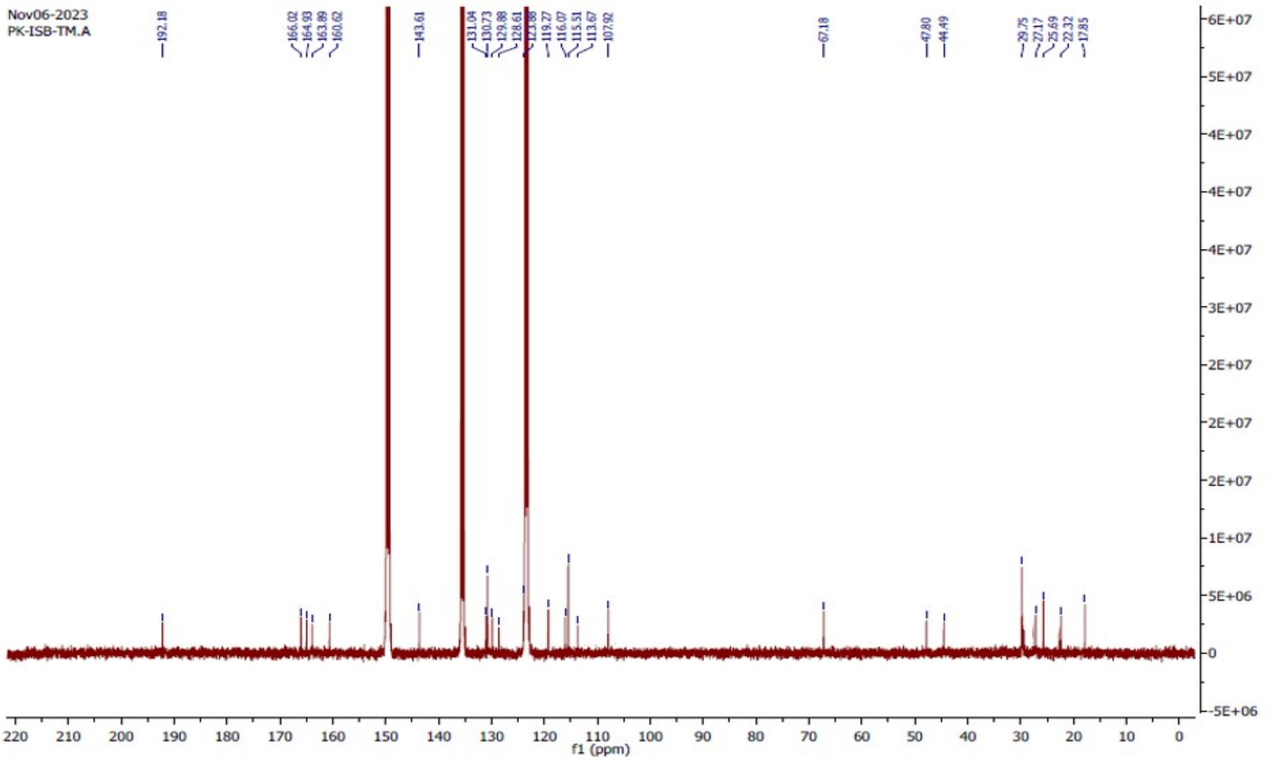

HRMS of IBC-10

Elemental Composition Report **Page 1**

Single Mass Analysis
Tolerance = 100.0 PPM / DBE: min = -1.5, max = 50.0
Element prediction: Off
Number of isotope peaks used for i-FIT = 3

Monoisotopic Mass, Even Electron Ions
28 formula(e) evaluated with 1 results within limits (up to 3 closest results for each mass)
Elements Used:
C: 0-26 H: 0-100 N: 0-1 O: 0-5 S: 0-1

PK-ISB-TM
QMI DIVISION, CSIR-IIIM JAMMU
Xevo G2-XS QTOF YFC2015
11-Jan-2024
15:22:16
110124_06 5 (0.121)
1: TOF MS ES+
1.51e+007

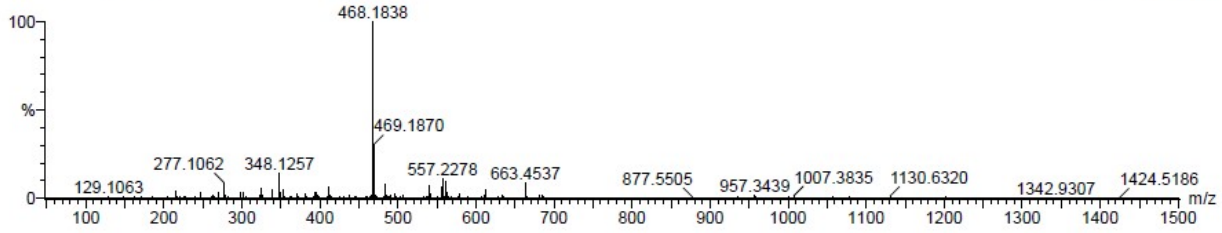

	Mass	Calc. Mass	mDa	PPM	DBE	i-FIT	Norm	Conf(%)	Formula
Minimum:					-1.5				
Maximum:			2.0	100.0	50.0				
	468.1838	468.1845	-0.7	-1.5	12.5	967.4	n/a	n/a	C26 H30 N O5 S

1a

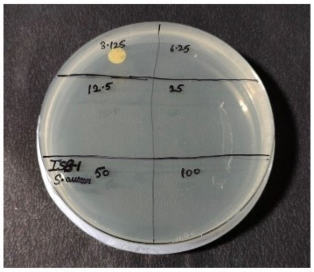

1b

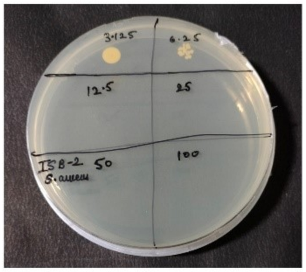

1c

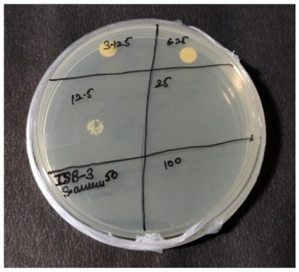

2a

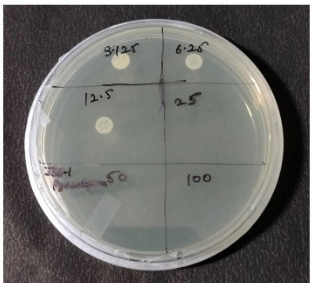

2b

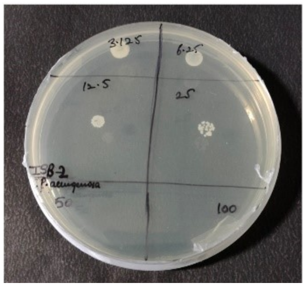

2c

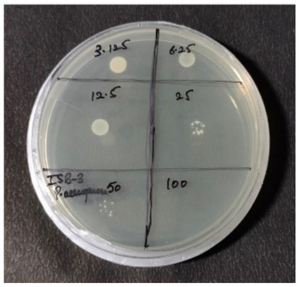

3

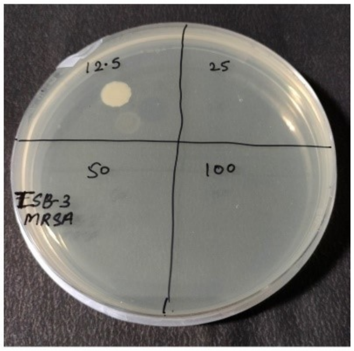

4

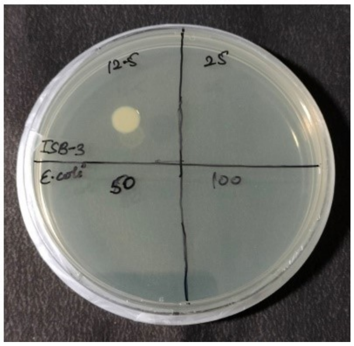

Figures- MBC of potent inhibitors against *S. aureus*, MRSA, *E. coli* and *P. aeruginosa*.

Figure 1a, 1b, 1c- MBC of **IBC, IBC-2** and **IBC-3** against *S. aureus*

Figure 2a, 2b, 2c- MBC of **IBC, IBC-2** and **IBC-3** against *P. aeruginosa*

Figure 3- MBC of **IBC-3** against MRSA

Figure 4- MBC of **IBC-3** against *E. Coli*
